# O-GlcNAcylation with ubiquitination stabilizes METTL3 to promoting HMGB1 degradation to inhibit ferroptosis and enhance gemcitabine resistance in pancreatic cancer

**DOI:** 10.1186/s10020-025-01285-4

**Published:** 2025-06-10

**Authors:** Qiuhong Wang, Dong Wei, Chunman Li, Xiawei Yang, Kun Su, Tao Wang, Renchao Zou, Lianmin Wang, Dongyun Cun, Bo Tang, Tao Wu

**Affiliations:** Departments of Hepatopancreatobiliary Surgery, The Second Affiliated Hospital of Kunming Medical University, No.374 Yunnan-Burma Road, Wuhua District, Kunming City, Yunnan Province 650101 China

**Keywords:** METTL3, Pancreatic cancer, HMGB1, O-GlcNAcylation, Ferroptosis

## Abstract

**Background:**

Pancreatic cancer is highly lethal, and METTL3 plays a crucial role in the regulation of m6A. However, the therapeutic potential of METTL3 in pancreatic cancer is unclear.

**Methods:**

By means of functional gain and loss experiments, the relationship between METTL3 and gemcitabine resistance as well as ferroptosis was explored. The target genes of METTL3 were screened, and the influence of O-GlcNAcylation on the stability and ubiquitination modification of METTL3 was dissected.

**Results:**

We found that METTL3 is highly expressed in pancreatic cancer and regulates ferroptosis and gemcitabine resistance by promoting the degradation of HMGB1 in an m6A-YTHDF2-dependent manner. O-GlcNAcylation modulates the METTL3’s stability. The interaction between the deubiquitinase EIF3H and METTL3 is influenced by O-GlcNAcylation.

**Conclusion:**

METTL3 affects pancreatic cancer progression by promoting degradation of HMGB1. The O-GlcNAcylation of METTL3 not only stabilizes METTL3 but also augments EIF3H-mediated deubiquitination. These findings uncover potential mechanisms underlying gemcitabine resistance in pancreatic cancer and offer potential therapeutic strategies for the treatment of this disease.

**Supplementary Information:**

The online version contains supplementary material available at 10.1186/s10020-025-01285-4.

## Introduction

Pancreatic cancer is a malignant tumor related to the gastrointestinal system (Balachandran et al. 2019), typically presenting at an advanced metastatic stage, rendering it unresectable and exhibiting limited response to chemotherapy (Maitra and Topol 2024). Surgical resection is not beneficial for over 80% of patients (Smith et al. 2019), as those who undergo radical resection face a high risk of recurrence (Neoptolemos et al. 2018). In recent years, there has been a global increase in the prevalence and mortality rates of pancreatic cancer, with only a 12.8% 5-year survival rate (https://seer.cancer.gov/) observed. Gemcitabine (GEM)-based combined chemotherapy strategies remain the preferred treatment for advanced pancreatic cancer (Qi et al. 2023). However, both GEM monotherapy and GEM combined with chemotherapy have shown very limited efficacy in improving the survival rate of patients with advanced PDAC due to GEM resistance (Gu et al. 2021). Therefore, overcoming GEM resistance and identifying new targeted molecules against GEM are key challenges in the treatment of pancreatic cancer.

Ferroptosis is an iron-dependent form of cell death characterized by excessive lipid peroxidation (Chen et al. 2021a, b). It primarily manifests as mitochondrial abnormalities, iron accumulation, and lipid peroxidation leading to plasma membrane rupture (Tang et al. 2021). Recent studies have demonstrated the association between ferroptosis and GEM chemotherapy resistance in various tumor types, including pancreatic cancer (Qi et al. 2023). Specific investigations have revealed that FBW7 enhances the cytotoxic effect of gemcitabine by activating both ferroptosis and apoptosis (Ye et al. 2021), while SLC38A5 regulates ferroptosis to overcome gemcitabine resistance in pancreatic cancer (Kim et al. 2023).In recent years, numerous studies have highlighted the role of HMGB1 in regulating cell ferroptosis (Han et al. 2024; Liu et al. 2024). There are reports suggesting that targeting the MCP-GPX4/HMGB1 axis effectively triggers immunogenic ferroptosis in pancreatic ductal adenocarcinoma (Li et al. 2024a, b). However, whether HMGB1 can be targeted to inhibit GEM resistance and promote ferroptosis for inhibiting the development of pancreatic cancer remains unexplored. We observed that the binding free energy between HMGB1 and GEM in the protein-molecule docking model is less than zero, indicating a potential interaction between HMGB1 and GEM as a target molecule. This suggests that targeting HMGB1 may promote ferroptosis in pancreatic cancer cells while inhibiting GEM resistance and preventing further progression of this disease.

O-GlcNAcylation is a reversible posttranslational modification of proteins (He et al. 2023), resulting from glucose metabolism via the hexosamine biosynthesis pathway (HBP), which integrates glucose, amino acids, fatty acids, and nucleotides (Wulff-Fuentes et al. 2021). The regulation of O-GlcNAcylation involves two enzymes: O-GlcNAc transferase (OGT) and O-GlcNAcase (OGA) (Ma et al. 2022). Since its discovery, O-GlcNAcylation has been implicated in various cellular functions including signal transduction, protein localization and stability, transcriptional control, chromatin remodeling, mitochondrial function, and cell survival. Dysregulation of the O-GlcNAc cycle is associated with the progression of diverse diseases such as diabetes mellitus and its complications as well as cancer and cardiovascular/neurodegenerative disorders (Chatham et al. 2021). In pancreatic cancer specifically, emerging evidence suggests that alterations in O-GlcNAcylation impact disease progression through modulation of different substrates. For instance: promotion of pancreatic tumor growth by regulating malate dehydrogenase 1 through O-GlcNAcylation has been reported (Zhu et al. 2022); facilitation of ferroptosis in mesenchymal pancreatic cancer cells via ZEB1’s O-GlcNAcylation has also been observed (Wang et al. 2022); additionally, O-GlcNAcylation-mediated stabilization of SIRT7 promotes pancreatic cancer progression by disrupting the SIRT7-REGγ interaction (He et al. 2022). Therefore, it is imperative to identify novel substrate proteins for targeted regulation of pancreatic cancer progression.

M6A is the most prevalent epigenetic modification in eukaryotic cells and governs diverse biological processes (Wang et al. 2020a, b, c). The three primary regulatory factors of M6A epigenetic alterations are the M6A methyltransferase (writer), M6A demethylase (eraser), and M6A recognition protein (reader) (Ping et al. 2014; Fu et al. 2014). METTL3 functions as an S-adenosylmethionine (SAM) binding protein and acts as a catalyst by utilizing its internal SAM-binding domain to transfer methyl from SAM to the adenine base of RNA, resulting in the production of S-adenosylhomocysteine (SAHWang et al. 2020a, b, c). Emerging evidence suggests that METTL3 plays a crucial role in cancer development, acting either as an oncogene or tumor suppressor gene (Zeng et al. 2020), for instance: METTL3-mediated m6A modification of HDGF mRNA promotes gastric cancer progression (Wang et al. 2020a, b, c). Wang et al. also reported overexpression of METTL3 in breast cancer and identified BCL2 as a target of METTL3. They demonstrated that elevated m6A modification in Bcl-2 mRNA promoted its translation and ultimately facilitated cancer cell proliferation (Wang et al. 2020a, b, c). METTL3 facilitates m6A modification of suppressor of cytokine signaling 2 (SOCS2) and promotes its mRNA degradation through a YTHDF2-dependent pathway, thereby regulating the progression of liver cancer (Chen et al. 2018). Studies have demonstrated that elevated levels of METTL3 expression are correlated with advanced pathological stages in PDAC (Xia et al. 2019), as well as resistance to chemotherapy and radiotherapy in pancreatic cancer cells (Taketo et al. 2018). However, the underlying mechanisms remain elusive. Therefore, comprehending the molecular mechanisms and downstream targets governed by METTL3 in pancreatic cancer regulation may offer novel avenues for the treatment and diagnosis of this disease.

In this study, we investigated the molecular mechanism underlying the role of METTL3, an M6A WRITER, in pancreatic cancer development and its impact on gemcitabine sensitivity. Our findings demonstrate that METTL3 interacts with OGT and undergoes O-GlcNAcylation, thereby elucidating the specific modification site involved in O-GlcNAcylation of METTL3. This post-translational modification stabilizes METTL3 expression by enhancing its interaction with EIF3H, a deubiquitinating enzyme. Additionally, we discovered that METTL3 promotes HMGB1 degradation through a m6A-YTHDF2-dependent pathway, inhibits ferroptosis in pancreatic cancer cells, and confers resistance to gemcitabine treatment. Overall, our study uncovers a novel regulatory axis involving OGT-METTL3-HMGB1 that governs pancreatic cancer development and highlights the potential therapeutic strategy targeting ferroptosis and gemcitabine.

## Methods

### Cell lines, cell cultures and transfer

The PANC1 and BXPC3 cell lines, along with the HEK293T cell line, were acquired from a Chinese cell bank situated in Shanghai. These cellular cultures were maintained using Dulbecco‘s modified Eagle’s medium (DMEM, KeGene Bio, China) supplemented with 10% fetal bovine serum (FBS), along with penicillin at a concentration of 100 U/ml and streptomycin at a concentration of 100 µg/ml. The incubation process involved keeping the cells at a temperature of 37℃ within an environment saturated with humidity and containing approximately 5% CO_2_. In order to achieve transient expression of plasmids within HEK293T cells, we employed PEI transfection solution based on findings reported by Zhou et al. (2022). When introducing plasmids into various cancerous cell lines, Lipofectamine™ 3000 manufactured by Invitrogen was utilized according to guidelines provided by its manufacturer. The siRNA sequences: siYTHDF2: 5′-GCACAGAAGTTGCAAGCAA − 3′; siOGT: 5′-GCCUGAUAGAUCUGGCAAUTT-3′; siEIF3H: 5ʹ-GCAACTCTTGGAAGAAATATA-3ʹ.

PugNAc (ab144670) was purchased from Abcam; Cycloheximide (S7418) was purchased from Selleck. Actinomycin D (50-76-0) was purchased from MCE.

### Clinical samples

The tumor tissue and matched non-tumor tissue samples of pancreatic cancer were obtained from 7 patients who underwent surgery at The Second Affiliated Hospital of Kunming Medical University. All patients provided informed consent and did not receive any preoperative chemotherapy or radiotherapy. This study was approved by the The Second Affiliated Hospital of Kunming Medical University Ethics Review Board.

### Cell viability assay using CCK-8

The Cell Counting Kit-8 (CCK-8) (B34302, Bimake) was used according to the manufacturer’s instructions. To ensure clarity in experimental conditions, we have now explicitly listed all reagents and their specific concentrations. Specifically, for the cell viability assays using Gemcitabine, the treatment concentrations of Gemcitabine were specified as 0, 0.5, 1, 1.5, 2, and 2.5 µM, and these details have been updated in the corresponding figure legends to ensure transparency and reproducibility.

Briefly, cells were seeded in 96-well plates at a density of 1 × 10^4 cells per well. For the gemcitabine treatment groups, cells were treated with various concentrations of gemcitabine (0, 0.5, 1, 1.5, 2, and 2.5 µM) for a specified duration of 24 h. After the treatment period, 10 µL of CCK-8 reagent was added to each well and incubated in a 5% CO_2_ incubator at 37 °C for 2 h. The absorbance was then measured at 450 nm using a microplate reader. This procedure was followed consistently across all experiments to ensure comparability and reproducibility of the results.

### Quantitative real time-PCR

Total RNA was extracted from cells using TRIzol reagent (Invitrogen, USA). The cDNA synthesis was performed by reverse transcribing the RNA with a reverse transcription kit (Bio-Bio Engineering (Dalian) Co., Ltd.). Real-time fluorescent quantitative PCR was conducted using the SYBR-Green PCR Master Mix kit (TOYOBO, Japan). Please refer to Supplementary Table 1 for the sequences.

### Western blot

Cells were harvested and disrupted using a solution containing SDS. The resulting proteins were separated by electrophoresis on an SDS-PAGE gel and subsequently transferred to a PVDF membrane. The PVDF membrane was then blocked with non-fat milk (5%). Specific primary antibodies were applied to the PVDF membrane for incubation, followed by secondary antibody treatment. Detection of proteins was achieved through chemiluminescence analysis. The following is the antibody information: anti-Flag (F1804) antibodies were purchased from Sigma (MO, USA); anti-β-actin (#4970), anti-O-GlcNAc (#9875) and anti-HMGB1(#3935)were obtained from Cell Signaling Technology (MA, USA), anti-eIF3h (ab60942) was obtained from Abcam (Cambridge, UK). anti-HA (sc-57592) antibodies were purchased from Santa Cruz biotechnology (TX, USA); anti-Myc (60003-2-Ig), anti- METTL3 (15073-1-AP) and anti-YTHDF2(24744-1-AP) were purchased from Proteintech. Please refer to Supplementary Table 2 for the antibody.

### Cycloheximide treatment

The corresponding cells were seeded onto 6-well plates and subjected to treatment with cycloheximide (20 mg/mL) for 0, 4, 8, and 12 h prior to collection. Total proteins were extracted and subsequently analyzed by Western blotting using the respective antibodies.

### mRNA stability analysis

Actinomycin D, a transcriptional inhibitor commonly employed for RNA stability detection, was administered to transfected cells at a concentration of 5 µg/ml. Subsequently, the cells were collected at designated time intervals and total RNA was extracted using TRIzol reagent. The relative expression levels of HMGB1 mRNA were assessed via qRT-PCR analysis.

### ME-ELISA

The EpiQuik™ m6A RNA Methylation Quantification Kit (Colorimetric) (Epigentek, USA) is utilized for the colorimetric determination of total m6A levels in pancreatic cancer cells. Specifically, 200 ng of RNA is immobilized onto capture antibodies in each well for subsequent detection. Following multiple incubation steps, the m6A content is quantified using a colorimetric method at 450 nm and calculated based on a standard curve.

### MERIP-qPCR

The MERIP-qPCR procedure was conducted following the guidelines provided by the EpiQuik ™ CUT&RUN m6A RNA Enrichment (MeRIP) Kit. In brief, an immunocapture solution was prepared by combining reagents in 0.2 ml PCR tubes and rotating them at room temperature for 90 min. Each tube received 10 µl of NDE (Nuclear Digestion Enhancer) and 2 µl of CEM (Cleavage Enzyme Mix), followed by a 4-minute incubation at room temperature. The tubes were then placed on a magnetic device until the solution became clear, which took approximately 2 min. After discarding the supernatant, samples underwent three washes with 150 µl of WB (Wash Buffer), followed by one wash with 150 µl of PDB (Protein Digestion Buffer). Subsequently, samples were mixed with 20 µl of Protein Digestion Solution and incubated at a temperature of 55 ◦C for a duration of 15 min using a thermocycler without a heated lid. RPS (RNA Purification Solution) and absolute ethanol were applied to the samples to resuspend and cleanse the RNA Binding Beads through vortexing. The resuspended beads were then subjected to treatment with Elution Buffer totaling13 µl, allowing for an incubation period at room temperature lasting for5 minutes to release RNA from the beads.Finally,13µlof each sample was transferred into new0.2 ml PCR tubes either for immediate use or storage at -20℃.

### RIPqPCR

The endogenous RNA was captured in the nucleus or cytoplasm through antibody or epitope labeling, followed by isolation of RNA-binding proteins from the bound RNA using immunoprecipitation. Cells were crosslinked with 1% formaldehyde and treated with RIPA buffer containing 150 mM NaCl, RNase, and protease inhibitors. Subsequently, cell lysis was performed using a solution consisting of 0.5% sodium deoxycholate, 0.1% SDS, 1% NP40, 1 mM EDTA, and 50 mM Tris (pH 8.0) for a duration of 30 min before centrifugation to collect the precipitates. The supernatant was then incubated four times with primary antibodies against METTL3 and YTHDF2 (or corresponding IgG antibodies), followed by addition of protein A/G glycosylated microspheres which were shaken for two hours. After washing the cells three times with RIPA buffer, RNA extraction was carried out following crosslinking procedures. Finally, quantitative RT-PCR was employed to detect the extracted RNA.

### RNA pulldown assays

The initial step involved the utilization of the MEGAscript T7 Transcription Kit from Thermo Scientific to transcribe the RNA. Subsequently, we employed the Pierce RNA 3′ End Desthiobiotinylation Kit (20, 163, Thermo Scientific) to label the ends of the amplified RNA with desthiobiotin. Lastly, we conducted RNA pulldown experiments using the Pierce Magnetic RNA–Protein Pull-Down Kit (20, 164, Thermo Scientific). Specifically, a mixture was prepared by combining 2 mg of protein lysates, 50 pmol of biotinylated RNAs, and 50 µL of streptavidin beads. Following three washing cycles and an incubation period, immunoblotting analysis was performed after subjecting the streptavidin beads to boiling.

### Luciferase reporter assay

The regions of HMGB1 mRNA containing the methylation sites of METTL3 were cloned into a pGL3 plasmid. The mutated sequence was synthesized by GenePharma, located in Shanghai, China. To quantify luciferase activity, we utilized the dual-luciferase reporter assay system from Promega based in the United States. Using a GloMax 20/20 Luminometer also provided by Promega, we determined the relative luciferase activity as indicated by the ratio between firefly luciferase activity and Renilla luciferase activity.

### Immunohistochemical (IHC) staining

For clinical specimens and mouse xenograft samples, paraffin embedding was carried out using the R.T.U. Vectastain Kit (from Vector Laboratories). Immunohistochemical staining was performed according to the instructions provided with the immunohistochemistry kit. All stainings were evaluated using quantitative imaging methods, where the percentage and intensity of immunostaining were recorded. The H-score was calculated using the following formula:

$$\begin{array}{l}\mathrm H-\mathrm{score}=\sum\,(\mathrm{PI}\times\mathrm I)\\\;=\;(\mathrm{Percentage}\;\mathrm{of}\;\mathrm{cells}\;\mathrm{with}\;\mathrm{weak}\;\mathrm{intensity}\;\times\;1)\\\;+\;(\mathrm{Percentage}\;\mathrm{of}\;\mathrm{cells}\;\mathrm{with}\;\mathrm{moderate}\;\mathrm{intensity}\;\times\;2)\\\;+\;(\mathrm{Percentage}\;\mathrm{of}\;\mathrm{cells}\;\mathrm{with}\;\mathrm{strong}\;\mathrm{intensity}\;\times\;3)\end{array}$$

Here, PI represents the percentage of positively stained cells among all cells, and I denotes the staining intensity.

Ki-67 (Proteintech: 27309-1-AP, China); anti-CD133 (Proteintech: 66666-1-Ig, China).

### Plasmid construction and lentiviral infection

All plasmids were designed to clone the corresponding cDNA into the corresponding expression vector to produce the desired protein. For shRNA and PCDH vectors, the culture supernatant of HEK 293T cells was collected 48–72 h after transfection for virus preparation and target cells were infected with 70% infection efficiency. PCDH lentivirus was used to construct METTL3 wild-type (WT) overexpression cell lines, while lentiCRISPR method was used to generate METTL3 knockout (KO) cell lines. In brief, we used BsmBI enzyme to linearize the lentiviral CRISPR vector and constructed the guide RNA (shRNA) into the lentiCRISPR V2 lentiviral expression vector. The shMETTL3 sequence: 5’-GCACTTGGATCTACGGAATCC-3’.

### Proliferation assay

The cellular proliferation was assessed using the EdU kit (provided by KeyGen Biotech Co., Ltd.) to quantify the EdU incorporation. After 48 h of transfection, cells were seeded at a density of 2 × 105 cells per well in a 6-well plate and exposed to 10 nM EdU for a duration of 12 h. Subsequently, the cells were fixed and permeabilized with 0.5% Triton X-100 for a period of 20 min. Following this, the Click-iT reaction cocktail was introduced, and the cells were incubated under dark conditions for approximately half an hour. After two washes with PBS, DAPI dye was applied as a counterstain for about ten minutes in darkness before observing them using fluorescence microscopy.

### Sphere-forming assay

The PC cells were cultured in serum-free DMEM/F12 medium supplemented with 2% B-27 (Gibco, USA, #17504044), heparin (4 µg/ml), epidermal growth factor (20 ng/ml) (R&D, USA, #236-EG-200), and fibroblast growth factor (20 ng/ml) (PEPROTECH, USA, #AF-100–18 C). DMEM/F12 medium with varying glucose concentrations was prepared by combining equal volumes of low-glucose DMEM medium (Gibco, USA) and F12 medium (Gibco, USA) containing d-glucose from Aladdin Industrial Corporation (#G116304). The cells were seeded at a density of 500 cells per well in ultra-low adhesion 6-well plates. After 7 days of incubation, the cultures were examined for sphericity using a phase contrast optical microscope.

### Flow cytometry

To perform staining, a total of 5 × 10^5^ cells were seeded into a U-shaped 96-well plate and incubated with 5 µl of each antibody at a temperature of 4 °C for a duration of 30 min. Following PBS washing, the cells were collected by centrifugation at a gravity force of 1000 g for 5 minutes. Subsequently, the cells were resuspended in 300 µl of PBS and subjected to flow cytometry analysis. The CD133/1-PE antibody was procured from MiltenyiBiotec located in Bergisch Gladbach, Germany.

### Mitochondrial superoxides measurements

The MitoSOX™ Red mitochondrial superoxide indicator for live-cell imaging (Invitrogen) was utilized to measure the accumulation of mitochondrial superoxide. The manufacturer’s protocol was followed for this purpose. Briefly, adherent cells were cultured on glass coverslips in a six-well plate. After the specified treatments, a 5 µM MitoSOX™ working solution (1 ml) was added to the cells on the coverslips. Subsequently, incubation of the cells at 37 °C in darkness took place for 10 min. Following this, gentle washing with warm buffer was performed three times. Mounting of coverslips in warm buffer facilitated imaging using a confocal microscope.

### Determination of intracellular ROS, malondialdehyde (MDA), and glutathione (GSH)

The CellROX™ Deep Green Reagent (Invitrogen, C10444) is employed in the experimental procedure to observe fluorescence microscopy and detect intracellular levels of ROS. Briefly, prior to treatment, samples are exposed to a 5 µM solution of the reagent for a duration of 30 min. Subsequently, after a post-treatment period lasting 0.5 h, cells are gathered for measurements of fluorescence intensity. The evaluation of Malondialdehyde (MDA) and Glutathione (GSH) production within the cells is conducted using MDA Detection Kit and GSH Assay Kit respectively, which are provided by Solarbio Science & Technology Co., Ltd., Beijing, China.

### Mitochondrial membrane potential measurements

Tetramethylrhodamine Ethyl Ester (TMRE) (manufactured by Beyotime) is a commonly employed fluorescent dye for labeling mitochondria in viable cells. As per the provided guidelines, an appropriate quantity of TMRE buffer is introduced to the cells, which are then incubated at 37 °C in a light-restricted environment for a duration of 20 min. Following this, the cells are rinsed with serum-free medium and subsequently observed using a fluorescence microscope.

### Intracellular iron assay

The concentration of ferrous ions within the cell was assessed by utilizing FerroOrange dye (#F374, Dojindo Laboratories). Cells were cultured and subjected to specific treatments. Subsequently, a diluted solution (1:2000, v/v) of FerroOrange Orange dye was added to the cells and incubated at 37 °C for 30 min. Fluorescent images were captured using a confocal microscope.

### Immunoprecipitation (IP) and co-immunoprecipitation (co-IP)

Cells were disrupted in Pierce IP buffer (Thermo Fisher) supplemented with protease and phosphatase inhibitors (Sigma). Following incubation with specified antibodies, the lysates were combined with protein A/G agarose beads (Thermo Fisher). For proteins carrying a tag, we employed immunomagnetic beads conjugated to anti-Flag/anti-HA antibody. Subsequent to immunoprecipitation, protein A/G agarose or magnetic beads underwent three washes using TBST (0.1% Tween-20, 150 mM NaCl, 10 mM Tris-HCl pH7.5), followed by elution in SDS lysis buffer (100 mM NaCl, 1% SDS, 50 mM Tris-HCl pH 7.5) for western blot analysis.

### In vivo ubiquitination assay

In ubiquitination experiments, cells were transiently transfected with the specified plasmids for 36 h, followed by treatment with 20 µM MG132 for an additional 8 h. The cells were then harvested. One-fifth of the cells were reserved for direct immunoblotting, while the remainder were processed for denaturing co-immunoprecipitation. The cells were lysed in denaturing buffer (described by Liu et al. 2023), and the lysates were incubated overnight HA-beads (Millipore; IP0010) at 4 °C. Subsequently, the beads were washed with buffers and Buffer C. Finally, the immunocomplexes were eluted using elution buffer, and the samples were analyzed by immunoblotting with indicated antibodies as detailed by Liu et al. (2023).

### Xenograft model

Our-week-old nude mice were purchased from Cavens, Changzhou, China. The nude mice were randomly allocated into 5 groups without any specific selection criteria. The mice were raised under specific pathogen-free conditions. Pancreatic cells (1 × 10^6^) stably expressing METTL3 and HMGB1 were implanted into the right flank of each mouse and allowed to grow, with 6 mice in each group (*n* = 6). Six days after cancer cell injection, gemcitabine (120 mg/kg, Sigma-Aldrich) was administered weekly for the gemcitabine treatment cohort, while RSL3 (5 mg/kg, Sigma-Aldrich) was injected once daily for the combined gemcitabine and RSL3 treatment group. Both gemcitabine and RSL3 were administered via intraperitoneal injection. This treatment regimen continued until the final observation week. The animal experimental protocol was approved by the Animal Ethics Committee of the Animal Care and Use Committee of the Ethical Institution of The Second Affiliated Hospital of Kunming Medical University. The investigators did not blind the animal experiments.

### Bioinformatical analysis

The survival curve of METTL3 in pancreatic cancer was obtained from the database https://smuonco.shinyapps.io/PanCanSurvPlot/; METTL3 differential genes and METTL3 peak genes were obtained from GSE146806 and GSE132306; METTL3 targeted genes were downloaded from the database http://rm2target.canceromics.org/#/home, and METTL3 differential genes in pancreatic cancer were obtained from UALCAN (uab.edu); ferroptosis-related genes were obtained from FerrDb (zhounan.org); the expression of related proteins such as METTL3 and YTHDF2 in pancreatic cancer was downloaded from Proteomic Data Commons (cancer.gov); Welcome to SRAMP, an online m6A site predictor (cuilab.cn) predicted HMGB1 m6A modification sites; the docking model of HMGB1 and gemcitabine was analyzed by autodockvina and visualized by Pymol.

### Molecular docking and interaction analysis

#### Software and parameter settings

All docking experiments were performed using AutoDock Vina, a widely recognized and validated tool for molecular docking simulations. Prior to docking, the target protein structures were prepared by removing water molecules, adding polar hydrogens, and assigning Gasteiger charges. Ligands were prepared using Open Babel and converted into the required format.

The docking parameters were carefully configured to ensure accurate prediction of binding affinities. The exhaustiveness parameter was set to 8 to balance computational efficiency with sampling accuracy. The grid box dimensions were determined based on experimentally validated ligand-binding sites obtained from the Protein Data Bank (PDB) entries and previously reported structural studies. These regions were further refined using cavity detection tools such as CASTp and SiteMap to ensure that the selected volume encompassed the most probable interaction interface. A grid spacing of 1 Å was used to achieve high-resolution docking results while maintaining computational feasibility.

#### Identification of key interaction residues

Following the docking simulations, we analyzed the resulting models to identify the most favorable binding modes between the interacting molecules. To further elucidate the nature of these interactions, key amino acid residues involved in hydrogen bonds, hydrophobic contacts, and other non-covalent interactions within the binding interface were identified and annotated. Visualization and analysis were conducted using PyMOL, which facilitated the detailed depiction of residue-level interactions and improved our understanding of the molecular basis of recognition.

#### Energy calculations and visualization

To evaluate the thermodynamic stability of the predicted protein-ligand complexes, binding free energy calculations were carried out using the MM-PBSA (Molecular Mechanics Poisson-Boltzmann Surface Area) method. This approach integrates molecular mechanics energies, solvation free energies, and surface area contributions to estimate the overall binding affinity. All MM-PBSA calculations were performed using the AmberTools package with optimized force fields for both proteins and ligands.

In addition, three-dimensional visualizations of the binding interfaces were generated using PyMOL software, enabling detailed spatial analysis of interaction patterns and the energetic contributions of specific residues. These visualizations provided valuable insights into the structural determinants of binding and helped interpret the docking outcomes more comprehensively.

### Statistical analysis

Data were collected from a minimum of three independent experiments. Statistical analysis was conducted using Prism software (GraphPad Prism 9.0.0). Unless otherwise stated, all data are presented as mean ± standard deviation. Paired or unpaired Student’s t-test (two-tailed) was employed for experiments involving two groups only. One-way ANOVA with multiple comparisons was utilized for experiments with more than two groups. Two-way ANOVA with multiple comparisons was applied for comparing four or more groups in a two-factor experiment. The level of statistical significance was set at *p < 0.05, ** *p* < 0.01 to determine significant differences among the experimental groups.

## Results

### METTL3 is upregulated in pancreatic cancer and promotes pancreatic cancer cell proliferation, stemness, and gemcitabine resistance

**Fig. 1:**
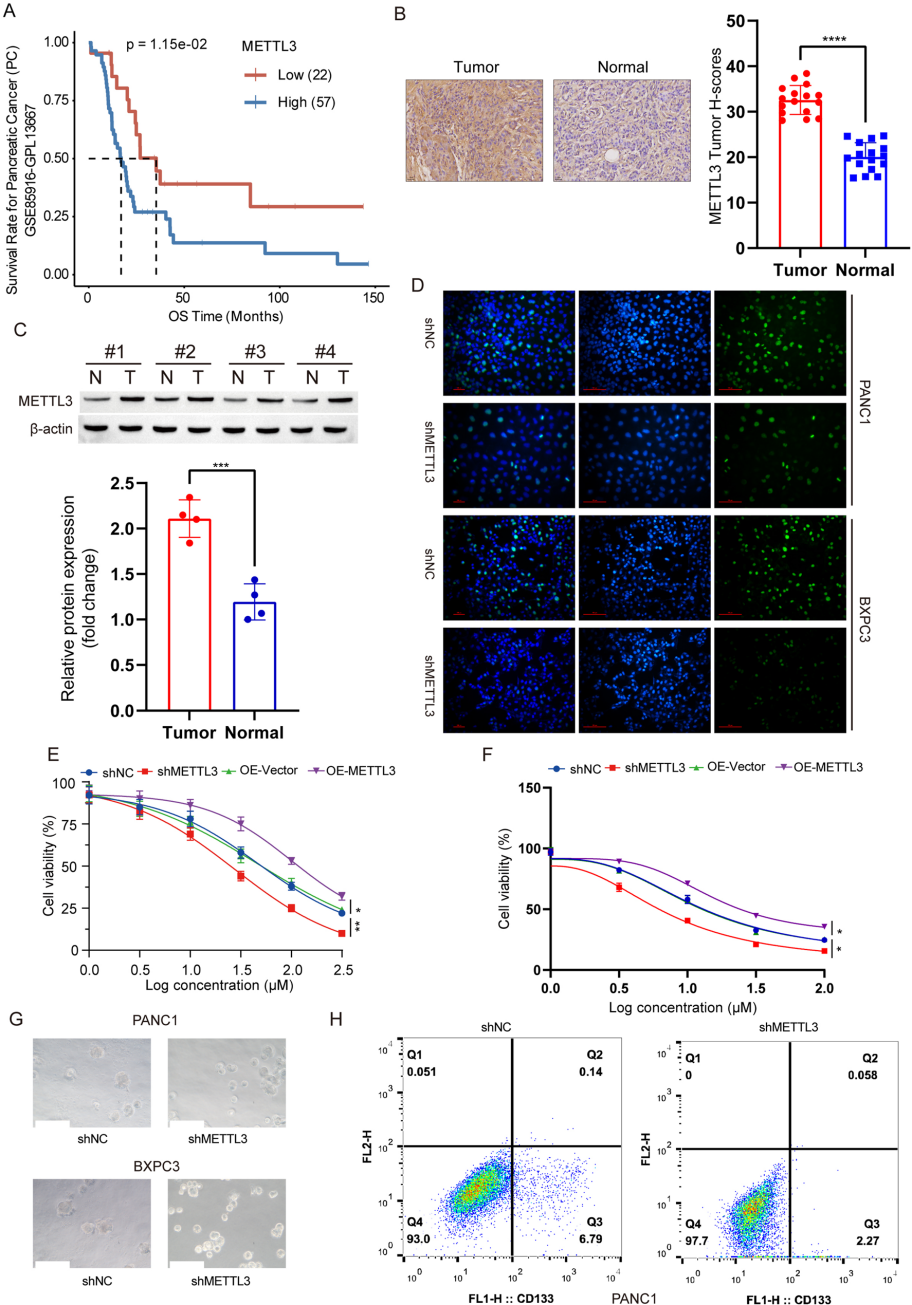
METTL3 is upregulated in pancreatic cancer and promotes pancreatic cancer cell proliferation, stemness, and gemcitabine resistance. **A** METTL3-related survival curves of pancreatic cancer patients. Data were obtained from the PanCanSurvPlot database. Kaplan-Meier survival analysis was performed to compare overall survival between high and low METTL3 expression groups. **B** Immunohistochemical analysis of METTL3 expression in pancreatic cancer tissues. Representative images show METTL3 staining intensity in tumor tissues and adjacent normal tissues. **C** Western blot detection and quantification of METTL3 expression in four pairs of pancreatic cancer tissues and matched adjacent normal tissues. Protein levels were normalized to β-actin as a loading control. Quantification was performed using ImageJ software. **D** EDU assay to assess cell proliferation in pancreatic cancer cell lines PANC1 and BXPC3 after METTL3 knockout. After 48 h, EDU incorporation was measured by fluorescence microscopy. Data are presented as mean ± SD (*n* = 3). **E** Sensitivity of PANC1 cells with stable knockout of METTL3, stable overexpression of METTL3, and their corresponding controls to gemcitabine. Cell viability was assessed using the CCK-8 assay after treatment with increasing concentrations of gemcitabine (0, 0.5, 1, 1.5, 2, 2.5 µM) for 48 h. IC50 values were calculated. **F** Sensitivity of BXPC3 cells with stable knockout of METTL3, stable overexpression of METTL3, and their corresponding controls to gemcitabine. Experimental conditions and analyses were identical to those described in panel **E**. **G** Cell spheroid assay to evaluate the effect of METTL3 knockout on the stemness of pancreatic cancer cell lines PANC1 and BXPC3. Spheroid formation was monitored under serum-free culture conditions for 7 days. Images were captured using a light microscope, and spheroid diameter was quantified. **H** Percentage of CD133 + pancreatic cancer cells after METTL3 knockout. Flow cytometry was used to quantify the CD133 + cell population. Cells were stained with anti-CD133 antibody and analyzed using a BD FACSCalibur flow cytometer. Data are presented as mean ± SD (*n* = 3). ***p < 0.001, **p < 0.01, *p < 0.05, ns *p* > 0.05

To investigate the impact of METTL3 expression on the prognosis of pancreatic cancer patients, we utilized the PanCanSurvPlot database and observed that patients with high METTL3 expression exhibited a poor prognosis (Fig. 1A). We collected multiple samples of pancreatic cancer tissues along with adjacent normal tissues and detected an upregulation in METTL3 expression in tumor tissues through qPCR analysis (Fig. S1A). Furthermore, immunohistochemistry and Western blot analyses confirmed a significant upregulation of METTL3 expression in tumor tissues compared to normal tissues (Fig. 1B, C). In order to clarify the role of METTL3 in the progression of pancreatic cancer, we established stable knockdown and overexpression models of METTL3 in two pancreatic cancer cell lines: PANC1 and BXPC3 (Fig. S1B). Utilizing the CCK8 assay, we discovered that overexpression of METTL3 promoted proliferation in both PANC1 and BXPC3 cells, while knockdown of METTL3 inhibited their growth (Fig. S1C, D). Furthermore, we observed a decrease in the number of EdU-positive cells in PANC1 and BXPC3 upon knockdown of METTL3 compared to the control group (Fig. 1D). Gemcitabine is currently considered as the first-line treatment for pancreatic cancer; however, resistance to gemcitabine has emerged as a major obstacle. Interestingly, our findings indicate that lower levels of METTL3 exhibit greater sensitivity to gemcitabine compared to higher levels of METTL3 (Fig. 1E, F). Given that cell pluripotency has been linked to GEM resistance (Hermann et al. 2007), we further investigated the impact of METTL3 on pancreatic cancer stemness. Initially, we conducted cell sphere formation experiments and observed a significant reduction in both volume of spheres formed by PANC1 and BXPC3 cells following METTL3 knockout (Fig. 1G; Fig. S1E). Additionally, flow cytometry analysis revealed a notable decrease in the percentage of CD133 + cells - a marker for stemness - upon METTL3 knockout in PANC1 and BXPC3 cells (Fig. 1H; Fig. S1F).

### The inhibition of ferroptosis in pancreatic cancer cells is mediated by METTL3

**Fig. 2:**
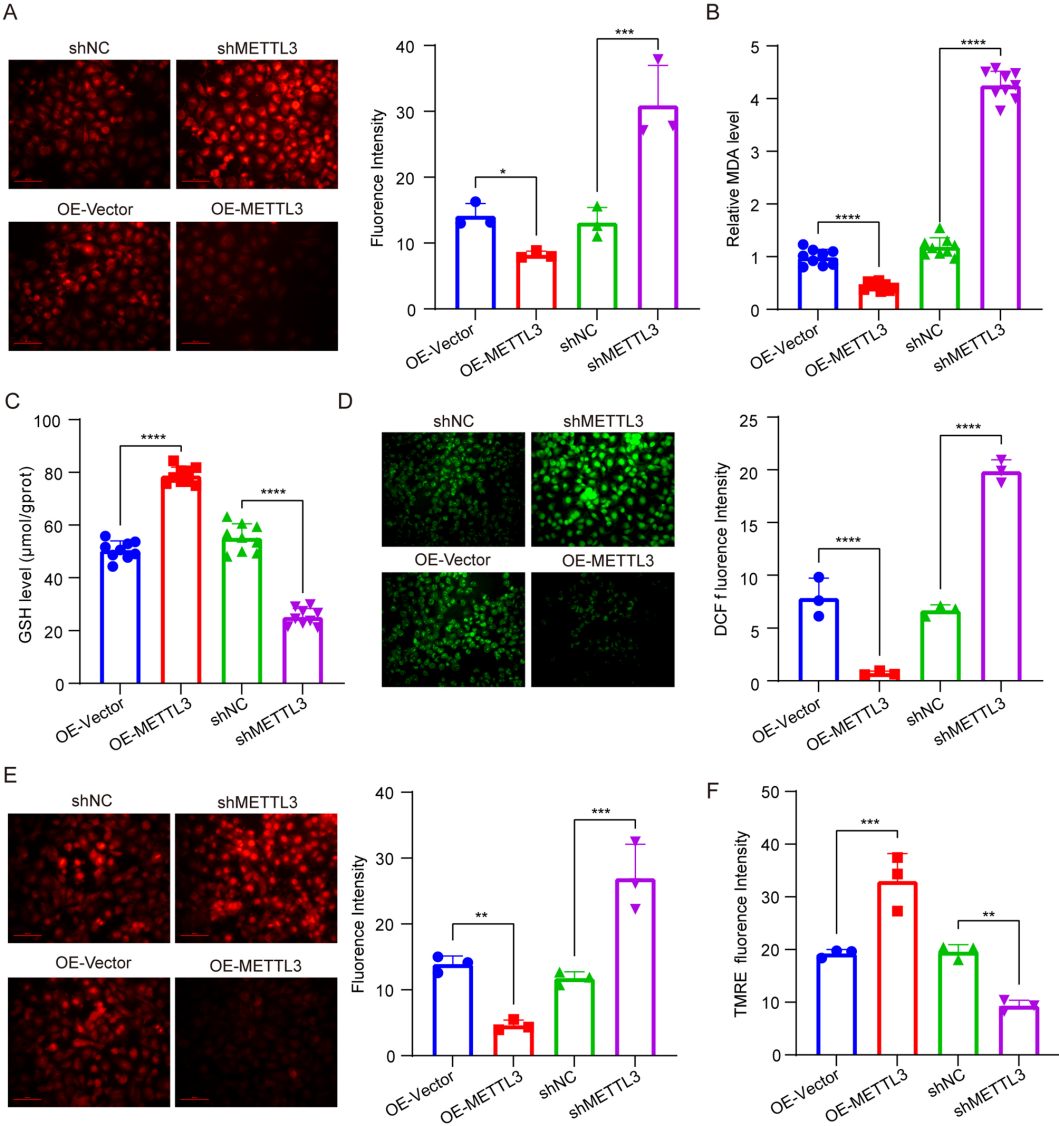
The inhibition of ferroptosis in pancreatic cancer cells is mediated by METTL3. **A** Representative images and quantification of mitochondrial superoxide levels in PANC1 cells with stable knockdown or overexpression of METTL3. Superoxide levels were measured using MitoSOX Red dye. Fluorescence intensity was quantified using ImageJ software. **B** Measurement of malondialdehyde (MDA) levels in PANC1 cells with stable knockdown or overexpression of METTL3. MDA content was determined using a thiobarbituric acid reactive substances (TBARS) assay kit according to the manufacturer’s protocol. **C** Measurement of glutathione (GSH) levels in PANC1 cells with stable knockdown or overexpression of METTL3. GSH content was detected using a GSH/GSSG ratio assay kit following the manufacturer’s instructions. **D** Measurement and quantification of reactive oxygen species (ROS) levels in PANC1 cells with stable knockdown or overexpression of METTL3. ROS levels were assessed using DCFH-DA fluorescent probe. Fluorescence intensity was measured at excitation/emission wavelengths of 488/525 nm. **E** Detection and quantification of ferrous ion levels in PANC1 cells with stable knockdown or overexpression of METTL3 using FerroOrange dye. Ferrous ion levels were visualized by fluorescence microscopy, and fluorescence intensity was quantified using ImageJ software. **F** Measurement of mitochondrial membrane potential in PANC1 cells with stable knockdown or overexpression of METTL3. ***p < 0.001, **p < 0.01, *p < 0.05, ns *p* > 0.05

To further elucidate the molecular mechanism underlying METTL3-mediated regulation of pancreatic cancer progression, we retrieved the GSE146806 and GSE132306 datasets from the GEO database. Subsequently, a KEGG analysis was performed on the overlapping genes between the top 2,000 differentially expressed genes identified in GSE146806 and the genes corresponding to METTL3 binding RNA peaks in GSE13,306. Our findings revealed that METTL3 is involved in regulating ferroptosis (Fig. S2A). Consequently, knockdown of METTL3 in stable knockout and stable overexpression models of PANC1 and BXPC3 led to an elevation in mitochondrial superoxide levels (Fig. 2A, Fig. S3A), increased MDA production (Fig. 2B, Fig. S3B), reduced GSH levels (Fig. 2C, Fig. S3C), accompanied by augmented ROS levels (Fig. 2D, Fig. S3D) and iron ion concentrations (Fig. 2E, Fig. S3E), as well as decreased mitochondrial membrane potential levels (Fig. 2F, Fig. S3F). Conversely, overexpression of METTL resulted in an opposite phenotype. Collectively, these results indicate that METTL inhibits cell ferroptosis and consequently promotes pancreatic cancer progression.

### HMGB1 was identified as a downstream target of METTL3

**Fig. 3:**
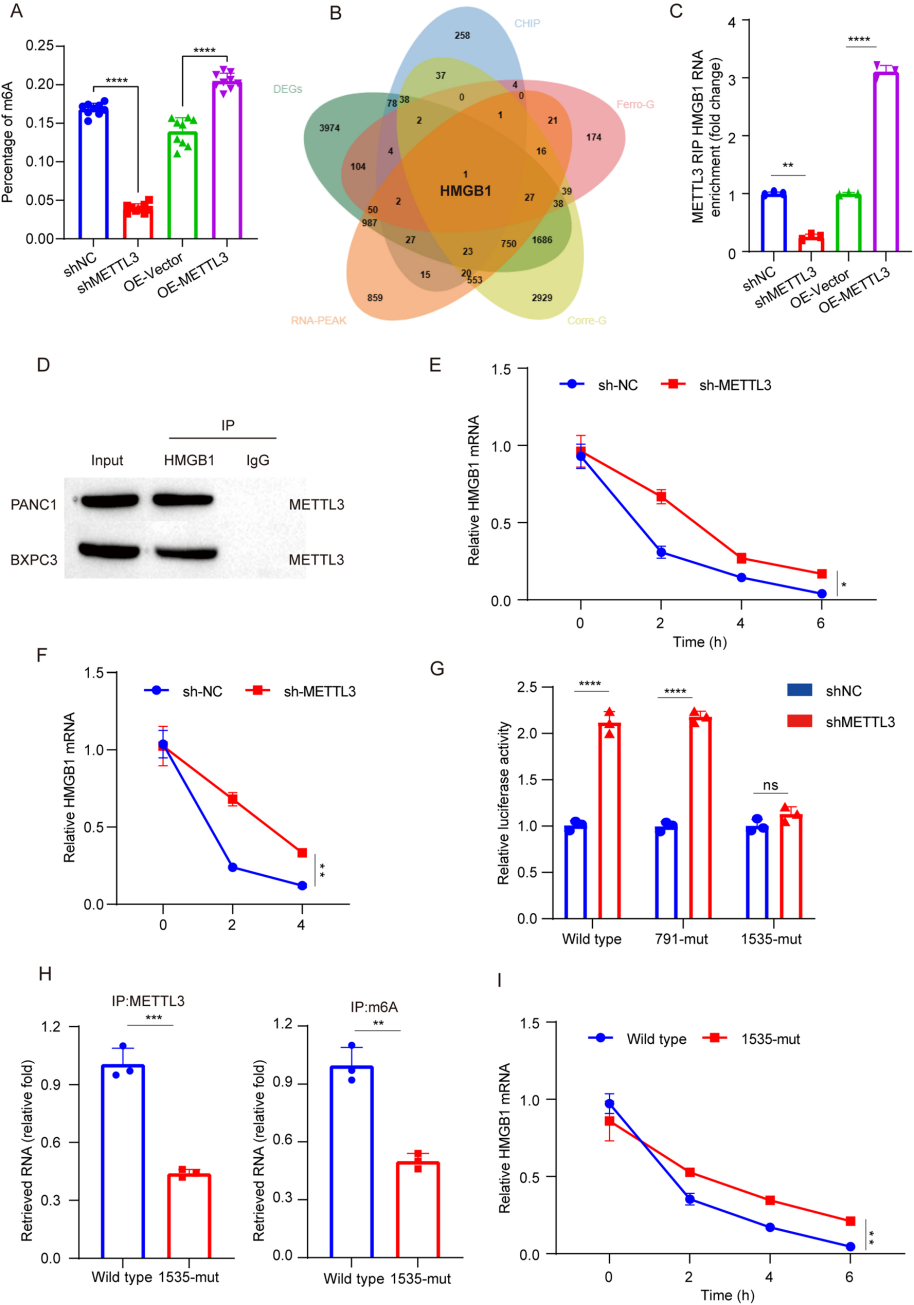
HMGB1 was identified as a downstream target of METTL3. **A** Quantitative determination of m6A levels in PANC1 cells with stable knockout and overexpression of METTL3. Total RNA was isolated, and m6A levels were measured using an m6A RNA Methylation Quantification Kit according to the manufacturer’s protocol. Data are presented as mean ± SD (*n* = 9). **B** Venn diagram showing overlapping genes obtained from the following analyses: differential genes from GSE146806, METTL3-correlated genes in pancreatic cancer from UALCAN (https://ualcan.path.uab.edu/index.html), target genes from the RM2Target database, and genes corresponding to METTL3-binding RNA peaks from GSE13306. The intersection identifies HMGB1 as a key downstream target. **C** RIP-qPCR validation of METTL3-regulated HMGB1 m6A modification in PANC1 cells. METTL3-bound RNA was immunoprecipitated using an anti-METTL3 antibody, and HMGB1 mRNA enrichment was quantified by qPCR. IgG was used as a negative control. Data are presented as mean ± SD (*n* = 3). **D** RNA pull-down experiments demonstrating the interaction between METTL3 and HMGB1 mRNA in PANC1 and BXPC3 cells. Biotin-labeled HMGB1 RNA probes were used to pull down METTL3 protein, which was detected by Western blot. A nonspecific RNA probe served as a negative control. **E** Deletion of METTL3 enhances the stability of HMGB1 mRNA in PANC1 cells. Cells were treated with actinomycin D (5 µg/mL) to inhibit transcription, and HMGB1 mRNA levels were measured at different time points (0, 2, 4, 6 h) by qPCR. Data are presented as mean ± SD (*n* = 3). **F** Deletion of METTL3 enhances the stability of HMGB1 mRNA in BXPC3 cells. Experimental conditions and analyses were identical to those described in panel **E**. **G** Relative luciferase activity of HMGB1 3’UTR with wild-type or mutated m6A sites after METTL3 silencing in PANC1 cells. Luciferase reporter plasmids containing either wild-type or mutated m6A sites in the HMGB1 3’UTR were transfected into cells. Luciferase activity was measured 48 h post-transfection. Data are presented as mean ± SD (*n* = 3). **H** The m6A level and METTL3 binding level of HMGB1 mRNA when the m6A methylation sites on HMGB1 mRNA were mutated in PANC1 cells, detected by MeRIP-qPCR. Mutations were introduced into the m6A sites using site-directed mutagenesis. Enrichment of m6A-modified HMGB1 mRNA was quantified by qPCR. Data are presented as mean ± SD (*n* = 3). **I** Mutation of m6A methylation sites on HMGB1 mRNA contributes to the enhancement of HMGB1 mRNA stability in PANC1 cells. Cells were treated with actinomycin D (5 µg/mL), and HMGB1 mRNA levels were measured at different time points (0, 2, 4, 6 h) by qPCR. Data are presented as mean ± SD (*n* = 3). ***p < 0.001, **p < 0.01, *p < 0.05, ns *p* > 0.05

As METTL3 is an M6A methyltransferase, we firstly detected the effect of METTL3 on the overall m6A level in PANC1 and BXPC3 cells. The results showed that METTL3 significantly increased the overall m6A level (Fig. 3A, Fig. S4A). The above experiments have shown that METTL3 regulates ferroptosis in pancreatic cancer cells. In order to determine the downstream ferroptosis target regulated by METTL3, we used a Venn diagram showing differential genes obtained from GSE146806 analysis, correlation genes of METTL3 in pancreatic cancer obtained from UALCAN, target genes from RM2Target database, and the genes corresponding to METTL3 binding RNA peaks in GSE13306 and ferroptosis-related genes to obtain HMGB1 (Fig. 3B). The RIP-qPCR assay was employed to validate the binding of METTL3 in PDAC cells with either knockdown or overexpression. Overexpression of METTL3 led to an increased enrichment of HMGB1, whereas knockdown of METTL3 resulted in a significant decrease (Fig. 3C, Fig. S4B). RNA pull-down analysis demonstrated the direct interaction between full-length HMGB1 mRNA and the METTL3 protein (Fig. 3D). MeRIP-qPCR results revealed that the presence of METTL3 directly enhanced m6A modification on HMGB1 mRNA in PANC1 and BXPC3 cells (Fig. S4C, D). Furthermore, Western blotting showed that depletion of METTL3 elevated HMGB1 protein levels, while overexpression of METTL3 reduced them (Fig. S4E), which was further confirmed by qPCR analysis (Fig. S4F). Additionally, actinomycin D treatment was performed to assess the impact of METTL3 on HMGB1 mRNA stability, demonstrating that knockdown of METTL3 resulted in increased stability for HMGB1 mRNA (Fig. 3E, F). We referred to the SRMAP database (http://www.cuilab.cn/sramp) to determine the m6A sites, and found two highly confident m6A sites in the 3’ UTR. Next, we conducted a luciferase assay by constructing the 3’ UTR of HMGB1 (Fig. S4G) to evaluate the effect of METTL3 binding on transcription. In the mutant group, the adenine nucleotide at the m6A site was replaced with cytosine to eliminate the m6A modification. The relative luciferase activity (Firefly/ Renilla ratio) ) showed that METTL3 silencing enhanced the transcriptional activity of the 3’ UTR of HMGB1. It is noteworthy that the mutation at position 1535 eliminated the effect of METTL3 silencing, while the mutation at position 791 did not (Fig. 3G). The MeRIP-qPCR results further confirmed that position 1535 mediated the m6A methylation of HMGB1 mRNA (Fig. 3H, Fig. S4H) and the stability of HMGB1 mRNA (Fig. 3I, Fig. S4I).

### YTHDF2 mediates HMGB1 mRNA expression in an m6A-dependent manner

**Fig. 4:**
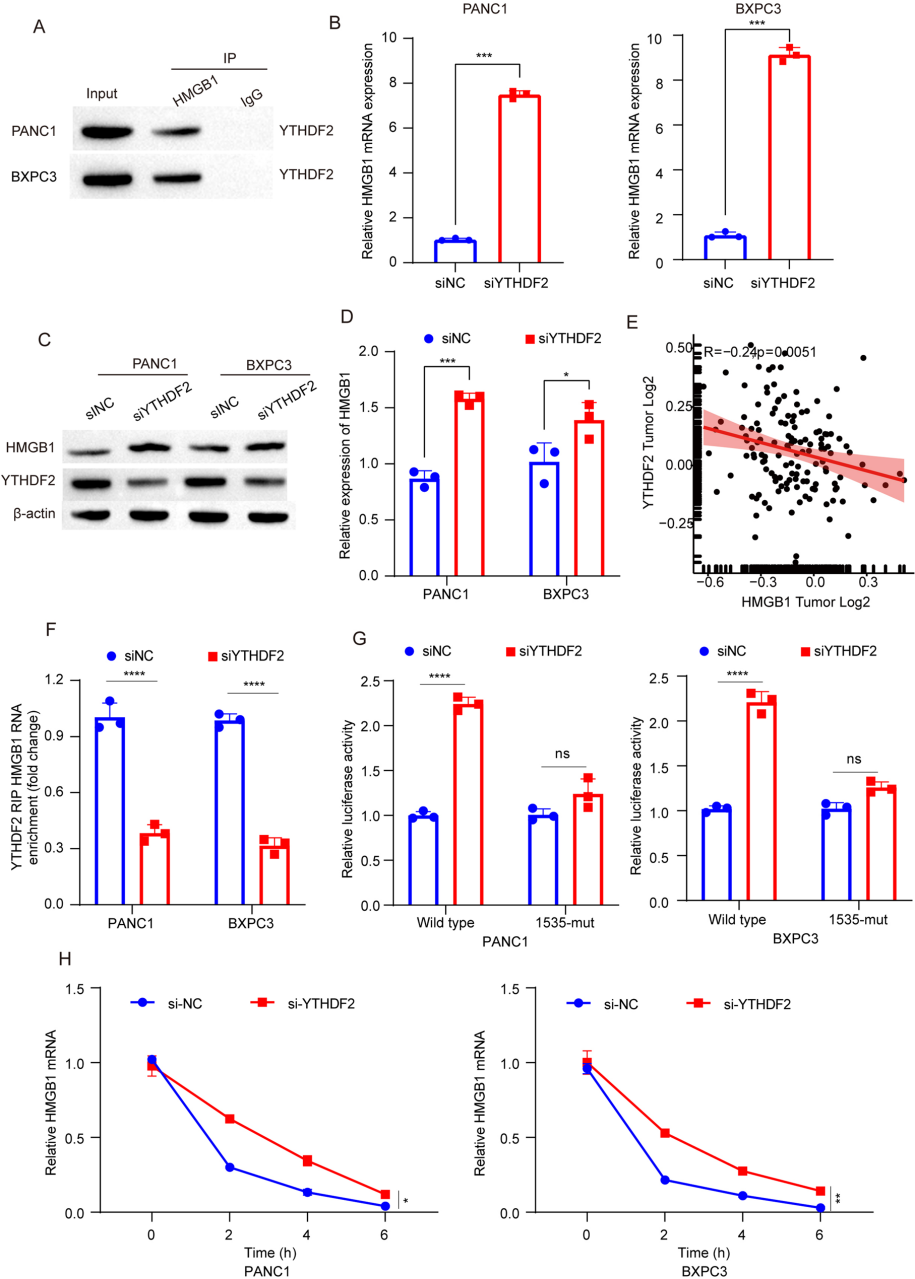
YTHDF2 mediates HMGB1 mRNA expression in an m6A-dependent manner. **A** RNA pull-down experiments demonstrating the interaction between YTHDF2 and HMGB1 mRNA in PANC1 and BXPC3 cells. Biotin-labeled HMGB1 RNA probes were used to pull down YTHDF2 protein, which was detected by Western blot. A nonspecific RNA probe served as a negative control. **B** mRNA levels of HMGB1 after interference with YTHDF2 in PANC1 and BXPC3 cells. YTHDF2 was knocked down using siRNA, and HMGB1 mRNA levels were measured by qPCR 48 h post-transfection. Data are presented as mean ± SD (*n* = 3). **C**, **D** Protein levels of HMGB1 after interference with YTHDF2 in PANC1 and BXPC3 cells. YTHDF2 was knocked down using siRNA, and HMGB1 protein levels were measured by Western blot 48 h post-transfection. β-actin was used as a loading control. Quantification was performed using ImageJ software. **E** Correlation between YTHDF2 and HMGB1 protein expression in pancreatic cancer. Immunohistochemical analysis was performed on pancreatic cancer tissues, and staining intensity was scored. Pearson correlation analysis was used to determine the relationship. **F** RIP-qPCR validation of YTHDF2-regulated HMGB1 m6A modification in PANC1 and BXPC3 cells. YTHDF2-bound RNA was immunoprecipitated using an anti-YTHDF2 antibody, and HMGB1 mRNA enrichment was quantified by qPCR. IgG was used as a negative control. Data are presented as mean ± SD (*n* = 3). **G** Relative luciferase activity of HMGB1 3’UTR with wild-type or mutated m6A sites after YTHDF2 silencing in PANC1 and BXPC3 cells. Luciferase reporter plasmids containing either wild-type or mutated m6A sites in the HMGB1 3’UTR were transfected into cells. Luciferase activity was measured 48 h post-transfection. Data are presented as mean ± SD (*n* = 3). **H**: Interference with YTHDF2 increases the stability of HMGB1 mRNA in PANC1 and BXPC3 cells. Cells were treated with actinomycin D (5 µg/mL) to inhibit transcription, and HMGB1 mRNA levels were measured at different time points (0, 2, 4, 6 h) by qPCR. Data are presented as mean ± SD (*n* = 3). ***p < 0.001, **p < 0.01, *p < 0.05, ns *p* > 0.05

The dynamic and reversible regulation of m6A modification is determined by the interaction between m6A writers and erasers. However, different downstream biological functions necessitate the recognition of m6A by distinct readers, including the regulation of m6A-modified transcripts. Stability analysis of HMGB1 mRNA revealed that METTL3 deletion (Fig. 3E, F) and mutation at the m6A site on HMGB1 mRNA (Fig. 3I, Fig. S4I) both enhanced mRNA stability. Previous studies have reported that YTHDF2 recognizes m6A modification to promote degradation of its target gene mRNA(Chen et al. 2022). Through RNA pull-down experiments and western blotting, we identified YTHDF2 as an m6A reader for HMGB1 mRNA in PANC1 and BXPC3 cells (Fig. 4A). qPCR experiments demonstrated a negative correlation between YTHDF2 expression and HMGB1 mRNA expression (Fig. 4B), which was further confirmed by western blotting showing increased levels of HMGB1 protein upon knocking down YTHDF2 (Fig. 4C, D). Analysis of protein expression data from the Proteomic Data Commons database revealed a negative correlation between YTHDF2 and HMGB1 protein expression in pancreatic cancer samples (Fig. 4E). Additionally, RIP-qPCR experiments showed that knocking down YTHDF2 led to increased levels of HMGB1 mRNA (Fig. 4F). Relative luciferase activity assays indicated that silencing YTHDF2 enhanced luciferase activity associated with HMGB1, while mutation at site 1535 abolished this effect caused by YTHDF2 silencing (Fig. 4G), providing further evidence for the role of knocking down YTHDF2 in enhancing stability of HMGB1 mRNA in PANC-1 and BXPC-3 cells (Fig. 4H). Our findings suggest a mechanistic control exerted by YTHDF2 on stability and expression levels of HMGB1 mRNA through an m6A-dependent manner.

### Inhibition of ferroptosis by METTL3 and gemcitabine resistance are mitigated by HMGB1

**Fig. 5:**
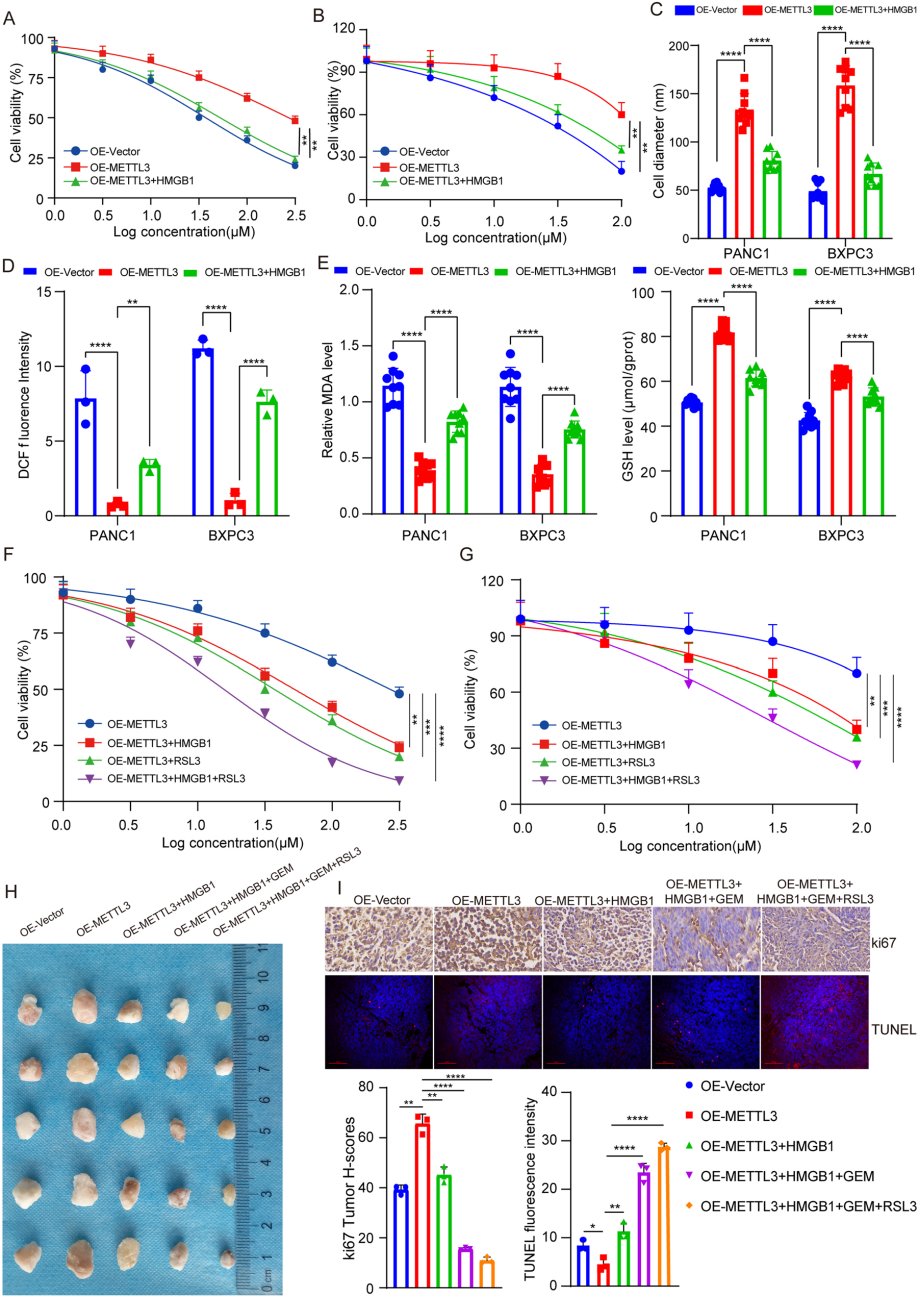
Inhibition of ferroptosis by METTL3 and gemcitabine resistance are mitigated by HMGB1. **A** Sensitivity to gemcitabine in PANC1 cells with stable overexpression of METTL3 and corresponding controls, with or without transfection of HMGB1. Cell viability was assessed using the CCK-8 assay after treatment with increasing concentrations of gemcitabine (0, 0.5, 1, 1.5, 2, 2.5 µM) for 48 h. IC50 values were calculated. Data are presented as mean ± SD (*n* = 3). **B** Sensitivity to gemcitabine in BXPC3 cells with stable overexpression of METTL3 and corresponding controls, with or without transfection of HMGB1. Experimental conditions and analyses were identical to those described in panel **A**. **C** Quantification of cell spheroid diameter in PANC1 and BXPC3 cells with stable overexpression of METTL3, transfected with or without HMGB1. Spheroid formation was monitored under serum-free culture conditions for 7 days. Images were captured using a light microscope, and spheroid diameter was quantified. Data are presented as mean ± SD (*n* = 9). **D** Quantification of reactive oxygen species (ROS) levels in PANC1 and BXPC3 cells with stable overexpression of METTL3, transfected with or without HMGB1. ROS levels were measured using the DCFH-DA fluorescent probe. Fluorescence intensity was measured at excitation/emission wavelengths of 488/525 nm. Data are presented as mean ± SD (*n* = 3). **E**: Quantification of malondialdehyde (MDA) and glutathione (GSH) levels in PANC1 and BXPC3 cells with stable overexpression of METTL3, transfected with or without HMGB1. MDA levels were measured using a thiobarbituric acid reactive substances (TBARS) assay kit, and GSH levels were determined using a GSH/GSSG ratio assay kit according to the manufacturer’s protocols. Data are presented as mean ± SD (*n* = 9). **F** Effect on cell viability of PANC1 cells with stable overexpression of METTL3 and corresponding controls, with or without transfection of HMGB1, treated with increasing concentrations of gemcitabine, with or without RSL3 (ferroptosis inducer) treatment. Cell viability was assessed using the CCK-8 assay. Data are presented as mean ± SD (*n* = 3). **G** Effect on cell viability of BXPC3 cells with stable overexpression of METTL3 and corresponding controls, with or without transfection of HMGB1, treated with increasing concentrations of gemcitabine, with or without RSL3 treatment. Experimental conditions and analyses were identical to those described in panel **F**. **H** Representative images of tumors after respective treatments. Tumor volume was measured using calipers, and tumor weight was recorded at the endpoint of the experiment. **I** Representative images of Ki67 and TUNEL staining in tumor tissues. Ki67 staining was used to assess cell proliferation, and TUNEL staining was used to detect apoptosis. Staining intensity was quantified using ImageJ software. ***p < 0.001, **p < 0.01, *p < 0.05, ns *p* > 0.05

Our previous studies have demonstrated that METTL3 inhibits ferroptosis and induces resistance to gemcitabine. HMGB1, a downstream target of METTL3 in the context of ferroptosis, the association between ferroptosis and gemcitabine resistance has been extensively documented in the literature. To investigate whether HMGB1 can regulate the sensitivity of pancreatic cancer cells to gemcitabine, we initially employed autodock vina software for analyzing the free energy between HMGB1 and gemcitabine molecules. The complexing free energy between HMGB1 and GEM was found to be -6.1 kcal/mol. Additionally, we visualized the docking model using pymol software (Fig. S5A), suggesting that HMGB1 may serve as a molecular target of GEM. We evaluated the impact of HMGB1 on GEM sensitivity in PANC1 and BXPC3 cells overexpressing METTL3. Our findings revealed that HMGB1 could enhance the sensitivity of METTL3-overexpressing cells towards GEM treatment (Fig. 5A, B). Similarly, HMGB1 could mitigate the effect of METTL3 on cell stemness by reducing sphere-forming cell diamater (Fig. 5C) and CD133^+^ cell populations (Fig. S5B). Subsequently, we investigated whether HMGB1 could alleviate the inhibition of ferroptosis caused by METTL3 in pancreatic cancer cells. For non-transfected cells, transfection with HMGB1 significantly reduced ROS levels (Fig. 5D) and MDA levels (Fig. 5E), while upregulating GSH levels (Fig. 5E). These results suggest that through alleviating ferroptosis inhibition and gemcitabine resistance induced by METTL3 overexpression, HMGB1 contributes to cellular responses in pancreatic cancer cells. To explore synergistic effects among gemcitabine treatment, ferroptosis induction, and HMBG1 expression on cell proliferation rates; we first assessed cell viability revealing that overexpression of HMBG1 combined with treatment involving both gemcitabine and RSL3 significantly reduced cell viability rates (Fig. 5F, G). The combined treatment of HMGB1 and GEM with RSL3 significantly suppressed tumor proliferation in in vivo experiments (Fig. 5H, Fig. S5C, D), immunohistochemistry also confirmed this result (Fig. 5I). These findings suggest that targeting HMGB1 in conjunction with GEM and ferroptosis represents a promising therapeutic strategy for pancreatic cancer.

### O-GlcNAcylation stabilizes METTL3 protein via enhancing the interaction between METTL3 and EIF3H

**Fig. 6:**
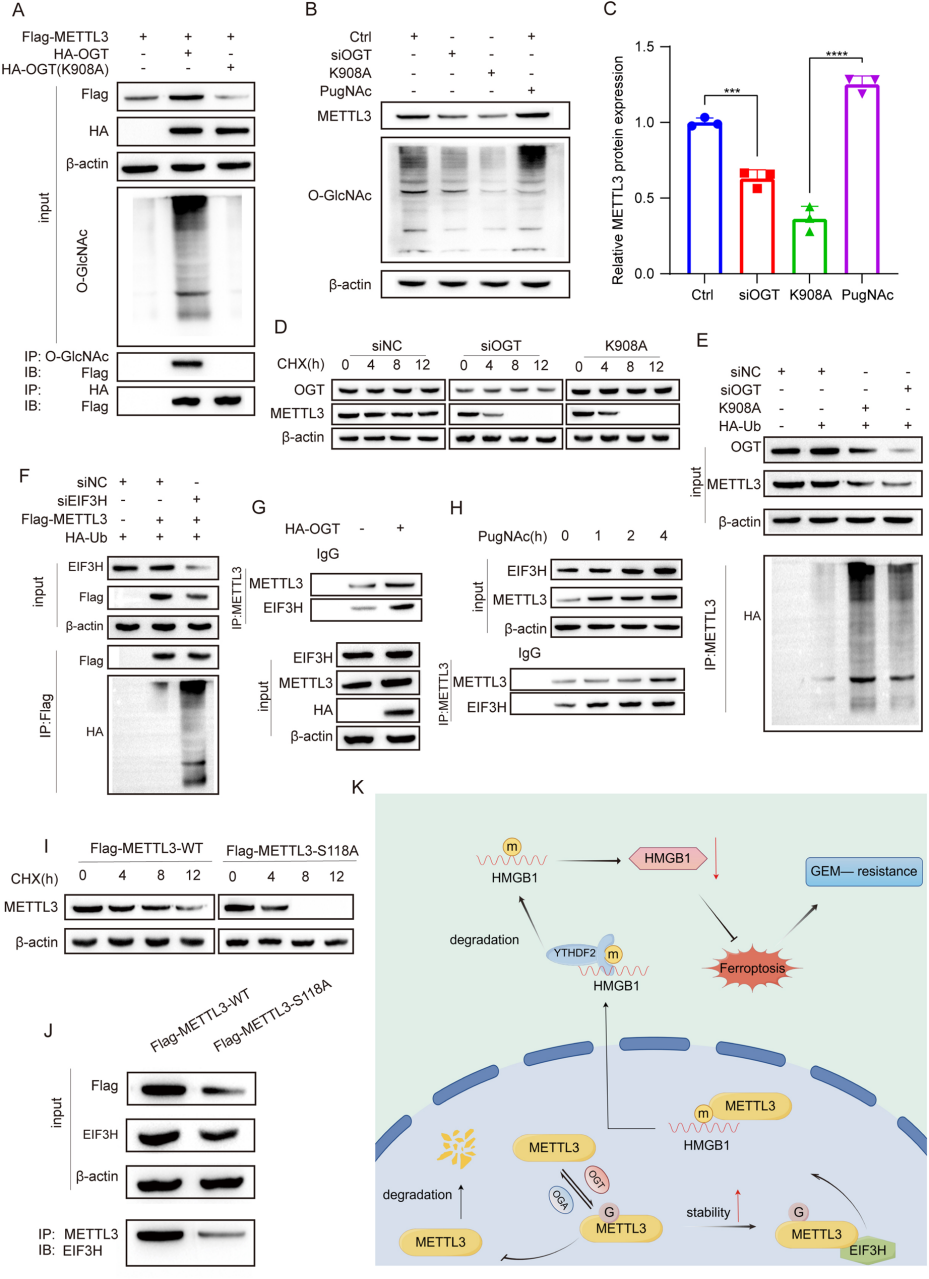
O-GlcNAcylation stabilizes METTL3 protein by enhancing the interaction between METTL3 and EIF3H. **A** Co-immunoprecipitation (Co-IP) assay was performed in HEK293T cells transfected with HA-OGT, HA-OGT-K908A, or Flag-METTL3 plasmids to detect protein interactions. Cell lysates were immunoprecipitated with anti-Flag antibody, followed by Western blotting with anti-HA and anti-Flag antibodies. **B** siNC, siOGT, or the K908A mutant were transfected into PANC1 cells, followed by treatment with or without the OGA inhibitor PugNAc (1 µM, 4 h). Cell lysates were subjected to immunoblotting using antibodies against METTL3, OGT, and loading control β-actin. **C** Western blot analysis was used to detect the expression level of METTL3 protein under corresponding treatments. Protein levels were normalized to β-actin as a loading control. Quantification was performed using ImageJ software. **D** siNC, siOGT, or the K908A mutant were transfected into PANC1 cells and treated with cycloheximide (CHX, 300 µg/mL) for designated time periods (0, 4, 8,12 h) to assess METTL3 protein stability. Protein levels were quantified using ImageJ software. **E** PANC1 cells treated with siNC, siOGT, or K908A and HA-Ubiquitin (HA-Ub) were immunoprecipitated with protein A/G agarose and incubated with anti-METTL3 antibodies, followed by HA Western blotting to detect ubiquitination levels. **F** PANC1 cells were transfected with siEIF3H or siNC, and METTL3 ubiquitination was detected by immunoprecipitation with anti-Flag antibody, followed by Western blotting with anti-Ubiquitin antibody. **G** PANC1 cells transfected with HA-OGT were immunoprecipitated with protein A/G agarose and incubated with anti-METTL3 antibodies. Western blotting for METTL3 and EIF3H was performed to assess their interaction. **H** PANC1 cells treated with PugNAc (1 µM) were immunoprecipitated with protein A/G agarose and incubated with anti-METTL3 antibodies. Western blotting for METTL3 and EIF3H was subsequently conducted to evaluate their interaction. **I** METTL3-WT or METTL3-S118A mutants were transfected into PANC1 cells and treated with CHX (300 µg/mL) for designated time periods (0, 4, 8, 12 h) to monitor METTL3 protein stability. Protein levels were quantified using ImageJ software. **J** METTL3-WT or METTL3-S118A mutants were transfected into PANC1 cells. Protein A/G agarose immunoprecipitation was performed with anti-METTL3 antibodies, followed by Western blotting for EIF3H to assess their interaction. **K** Schematic diagram illustrating the mechanism by which O-GlcNAcylation stabilizes METTL3 protein via enhancing the interaction between METTL3 and EIF3H. ***p < 0.001, **p < 0.01, *p < 0.05, ns *p* > 0.05

Our previous studies have demonstrated that high expression of METTL3 is associated with a poor prognosis in pancreatic cancer and promotes the progression of pancreatic cancer cells. In order to identify new molecular mechanisms regulating the function of METTL3, we discovered its involvement in lipid metabolism, central carbon metabolism, amino acid metabolism, and nucleic acid metabolism (Fig. S2A). Additionally, O-GlcNAcylation integrates glucose, amino acids, fatty acids, and nucleic acid metabolism (Wulff-Fuentes et al. 2021). As shown in the UALCAN database, OGT also shows a significant upregulation of protein expression in pancreatic cancer (Fig. S6A). Therefore, we hypothesized whether METTL3 undergoes O-GlcNAcylation mediated by OGT (O-GlcNAc Transferase). Initially, we obtained the 3D structure of the METTL3-METTL14 complex and OGT dimer from the PDB database (https://www.rcsb.org/), as well as the complex model of METTL3-METTL4 and OGT from Cluspro (Fig. S7A). The binding free energy analysis using revealed that the binding free energy between METTL3-OGT is less than 0 (Fig. S7B), suggesting an interaction between them. Co-IP experiment confirmed this interaction between METTL3 and OGT (Fig. 6A). Furthermore, while the K908A mutation in OGT eliminated its enzymatic function without affecting protein abundance according to previous research (Li et al. 2018), our study found that this mutant could still interact with METTL3. However, this mutant failed to induce O-GlcNAcylation of METTL3, suggesting that enzymatic activity of OGT is necessary for the process (Fig. 6A). To determine the region where OGT and METTL3 interact, we constructed a truncated model of OGT (Fig. S7C). We found that, except for the δTPR region, METTL3 could bind to it (Fig. S7D), indicating that METTL3 interacts with the OGT-TPR region. Since O-GlcNAcylation usually affects protein stability, we downloaded the expression of METTL3 and OGT proteins in pancreatic cancer patients from the Proteomic Data Commons and plotted a correlation curve, which showed that OGT protein expression and METTL3 protein expression were positively correlated in pancreatic cancer (Fig. S7E). In PANC1 cells, we detected the expression of METTL3 and the overall O-GlcNAcylation by interfering with OGT and giving PugNAc to inhibit OGA, and found that after inhibiting OGT, the expression level of METTL3 protein was lower, while inhibiting OGA could enhance METTL3 protein expression, and the expression of METTL3 was significantly downregulated after O-GlcNAcylation inhibition (Fig. 6B, C). However, O-GlcNAcylation did not affect the expression of METTL3 mRNA (Fig. S7F). Next, we detected the effect of O-GlcNAcylation on the stability of METTL3 protein. As shown in Fig. 6D, knocking down OGT and inhibiting O-GlcNAcylation promoted the degradation of METTL3. The ubiquitin-proteasome pathway mediates 80-85% of protein degradation (Li et al. 2023a, b). O-GlcNAcylation can prevent the degradation of target proteins by reducing their ubiquitination, and the underlying mechanism is to recruit deubiquitinating enzymes to O-GlcNAcylated proteins (Ma et al. 2022). The ubiquitination test also confirmed that knockdown of OGT and K908A mutations did enhance the level of METTL3 ubiquitination (Fig. 6E), further clarifying that OGT mainly mediates polyubiquitination in a K48-dependent manner (Fig. S7G). Next, we explored the mechanism by which O-GlcNAcylation regulates the stability of METTL3. We downloaded the binding protein of METTL3 (PXD036899) from the proteomexchange.org database, downloaded the interacting proteins of METTL3 and all deubiquitinating enzymes from the databases https://thebiogrid.org/ and https://iuucd.biocuckoo.org/index.php respectively, and obtained the only overlapping protein EIF3H through the Venn diagram (Fig. S7H). We verified that there was no correlation between EIF3H mRNA and METTL3 mRNA in the GEPIA database (Fig. S7I), but a positive correlation at the protein level (Fig. S7J). The above data illustrate that EIF3H regulates the expression of METTL3 protein. The ubiquitination experiment demonstrated that knockdown of EIF3H significantly enhanced the ubiquitination of METTL3 (Fig. 6F). Through Co-IP experimentation, we observed a binding interaction between EIF3H and METTL3(Fig. 6G). By analyzing the binding free energy of EIF3 and METTL3, as well as EIF3 and METTL3-OGT complexes using Cluspro, we discovered that the binding free energy of EIF3 and METTL3-OGT complexes was lower compared to that of EIF3 and METTL3 within a similar interaction area. This suggests that OGT has the ability to enhance the binding affinity between METTL3 and EIF3. The Co-IP results further confirmed this observation, demonstrating that the addition of OGT augmented the interaction between METTL3 and EIFH (Fig. 6G). Similarly, PugNAc treatment also enhanced the binding between METTL3 and EIF3H (Fig. 6H). We combined multiple databases (https://services.healthtech.dtu.dk/services/NetOGlyc-4.0/; https://services.healthtech.dtu.dk/services/DictyOGlyc-1.1/; https://www.oglcnac.mcw.edu/) to predict that the O-GlcNAcylation modification site of METTL3 is S118, and this site is highly conserved in mice, rats and humans. We produced a mutant of METTL3 (S118A). The IP assay also showed significantly lower levels of O-GlcNAcylation on S118A compared to WT (Fig. S7K), indicating that S118 is the main site of O-GlcNAcylation of METTL3. Compared with WT, the S118A mutant promoted the degradation of METTL3 (Fig. 6I), inhibited the binding of METTL3 and EIF3H (Fig. 6J), and the ubiquitination experiment also proved that the S118A mutation significantly enhanced the ubiquitination modification of METTL3 compared with WT (Fig. S7L). Concurrently, we conducted functional rescue experiments on the S118A mutant to verify its effects on HMGB1 degradation and ferroptosis phenotype. The results indicated that the S118A mutation led to an increase in mitochondrial superoxide levels (Fig. S8A), an increase in MDA production (Fig. S8B), a decrease in GSH levels (Fig. S8C), accompanied by an increase in ROS levels (Fig. S8D) and iron ion concentration (Fig. S8E), as well as a decrease in mitochondrial membrane potential levels (Fig. S8F). The findings suggest that O-GlcNAcylation promotes the stability of METTL3 by enhancing the interaction between METTL3 and EIF3H.

## Discussion

More and more evidence suggests that M6A modification plays a crucial role in various biological processes, especially in tumor formation and cancer progression. As the catalytic subunit of the M6A writer, METTL3 has been found to collaborate with YTHDF2 and promote HCC progression by degrading the mRNA of SOCS2 (Chen et al. 2018). In pancreatic cancer, it has also been proven that METTL3 modulates ID2 by m6A methylation to regulate pancreatic cancer cell stemness (Chen et al. 2023). Previous studies mainly focused on the role of METTL3 in mediating mRNA metabolism and tumor progression through its target gene m6A methylation regulation, however, the limited understanding of METTL3-specific functional regulation, especially in post-translational modification, still exists. Here, we first prove that METTL3 is highly expressed in pancreatic cancer and inhibits ferroptosis-induced gemcitabine resistance, and HMGB1 is identified as a downstream target of METTL3. Furthermore, the m6A-YTHDF2-dependent pathway is upregulated to decrease HMGB1. Targeting HMGB1, combined with gemcitabine and ferroptosis inducer RSL3, can significantly inhibit tumor proliferation. Furthermore, we prove that METTL3 exists in O-GlcNAcylation, and stabilizes its expression, the S118 site O-GlcNAcylation maintains the stability of the METTL3 protein, the specific mechanism lies in that O-GlcNAcylation interferes with ubiquitination, i.e., O-GlcNAcylation enhances the interaction between METTL3 and deubiquitinase EIF3H, resulting in a lower level of ubiquitination modification and enhanced stability of the METTL3 protein (Fig. 6K). These findings provide new insights into the treatment of pancreatic cancer.

In recent years, post-translational modification of METTL3 has attracted more and more attention from academia. SUMOylation of METTL3 at K177, K211, K212 and K215 sites might repress its methyltransferase activity for m6A RNA methylation. Subsequent SUMOylation of METTL3 promotes colony formation and tumor growth of human non-small cell lung cancer (NSCLC) H1299 cells (Du et al. 2018), which is caused by downstream gene dysregulation. Lacylation-driven METTL3-mediated RNA m6A modification plays an important role in promoting the immunosuppressive ability of tumor-infiltrating myeloid cells (Xiong et al. 2022). In addition, METTL3 can bind to USP5, and this binding is promoted by ERK-mediated phosphorylation. ERK-dependent METTL3 stabilization affects cellular mRNA m6A methylation which could contribute to tumorigenesis (Sun et al. 2020). The E3 ubiquitin ligase RNF113A mediates the ubiquitin/proteasome-dependent degradation of METTL3 through the K48-linked multi-ubiquitin chain, reducing the level of m6A modification to promote the inhibition of acute myeloid leukemia (Yang et al. 2023). Our study revealed that OGT induced O-GlcNAcylation of METTL3 by interacting with METTL3, increasing the stability of METTL3 protein. In addition, we also found that METTL3 was O-GlcNAcylated at Ser118 site and emphasized its role in crosstalk ubiquitination. The improvement of METTL3 stability led to the enhancement of the level of m6A modification of downstream target genes. These findings also further clarify the regulatory network of post-translational modification of proteins and m6A modification of RNA in the pathogenesis of pancreatic cancer.

Our study provides novel insights into the post-translational regulation of METTL3 by identifying O-GlcNAc modification as a previously unrecognized but critical mechanism for its stability and functional modulation. While prior research has extensively explored the roles of SUMOylation and phosphorylation in regulating METTL3’s stability, subcellular localization, and catalytic activity, our findings uncover a new dimension of METTL3 regulation (Li et al. 2024a, b; Zhang et al. 2020). Specifically, we demonstrate that O-GlcNAc modification at S118 stabilizes METTL3 by enhancing its interaction with EIF3H. This discovery not only enriches the landscape of METTL3 post-translational modifications but also highlights an additional layer of complexity in its regulatory network. In terms of functional pathways, while SUMOylation and phosphorylation primarily influence METTL3’s subcellular localization and enzymatic activity, our study reveals that S118 O-GlcNAcylation exerts its effects through a distinct mechanism—promoting the binding between METTL3 and EIF3H to maintain protein stability. This novel pathway establishes a direct link between cellular metabolic status (via O-GlcNAc modification) and RNA methylation processes, suggesting that METTL3 serves as a key node connecting metabolism with epigenetic regulation. Such a connection underscores the intricate interplay between cellular metabolic cues and gene expression control. From a therapeutic perspective, targeting METTL3 modifications has emerged as a promising strategy in cancer treatment, given its central role in regulating RNA methylation and downstream oncogenic pathways. Our findings extend this concept by proposing that modulating O-GlcNAc modification could serve as a viable approach to regulate METTL3 levels and influence ferroptosis, a form of regulated cell death implicated in cancer biology. This opens new avenues for developing targeted therapies against diseases associated with METTL3 dysregulation, such as malignancies driven by aberrant m6A methylation.

O-GlcNAcylation has been demonstrated to impact various functional activities of proteins, including stability, transcriptional activity, localization, and protein-protein interactions (Chatham et al. 2021). The presence of O-GlcNAcylation at Thr58 competes with phosphorylation, leading to enhanced protein stability and hindered degradation by proteasomes for c-MYC (Chou et al. 1995). Inhibition of O-GlcNAcase (OGA) activity results in prolonged half-life of YAP through the inhibition of SCF^β−TRCP^ E3-ubiquitin ligase (Zhang et al. 2017). Moreover, accumulating evidence suggests that elevated levels of O-GlcNAcylation are closely associated with pancreatic cancer progression, metastasis and recurrence, ECM remodeling, and immunotherapy resistance (Sharma et al. 2020; Shang et al. 2021).In our study on pancreatic cancer, we observed a regulatory relationship between METTL3 expression and the extent of O-GlcNAcylation modification. Specifically, enhancing O-GlcNAcylation of METTL3 through exogenous transfection of OGT increased its protein stability. Conversely, loss-of-function mutation in the catalytic domain of OGT (K908A) reduced METTL3 expression. Similarly, treatment with PugNAc (an inhibitor targeting OGA) upregulated METTL3 expression. Deficiency in O-GlcNAcylation shortened the half-life of METTL3 by promoting K48-linked ubiquitination. Additionally, loss-of-function mutation S118A resulted in enhanced stability of METTL3 by inhibiting its binding to EIF3H protein via abrogating the interaction with this partner molecule. Further investigations are required to determine whether degradation-induced regulation downstream phenotypes occur due to loss-of-O-GlcNAcylation caused by S118A mutation. Interestingly, the S118A mutant does not completely abolish O-GlcNAcylation of METTL3, thereby implying the existence of potential alternative O-GlcNAc sites.

While our study primarily focuses on S118 as a key O-GlcNAc modification site and its role in stabilizing METTL3 expression, the residual O-GlcNAc signal observed after the S118A mutation suggests the potential existence of other unidentified O-GlcNAc modification sites. To fully decipher all potential O-GlcNAc modification sites on METTL3 and their functional significance, future investigations will inevitably require the use of advanced mass spectrometry techniques combined with bioinformatics tools. These approaches will not only enable precise localization and identification of every O-GlcNAc modification site on METTL3 but also reveal the unique roles of each site in regulating METTL3 expression, interactions, and involvement in cellular processes through subsequent functional validation experiments. Therefore, we consider this to be an important direction for future research, aiming to construct a more complete and detailed O-GlcNAc modification map of METTL3 to further elucidate its biological functions and pathophysiological significance. Additionally, this systematic approach will provide a valuable reference framework for studying O-GlcNAc modifications on other protein.

High mobility group box 1 (HMGB1) is a non-histone chromatin-associated protein that is widely distributed in eukaryotic cells and plays a role in DNA damage repair and genomic stability maintenance (Wang and Zhang 2020). HMGB1 appears to have conflicting functions in the progression and treatment of cancer. On the one hand, HMGB1 may promote tumor formation, for example, by playing an important role in regulating mouse oval cell activation and liver cancer development related to inflammation (Pusterla et al. 2013). On the other hand, LPS induces pro-inflammatory cytokines (such as IL-1β, IL-6, and TNF-α) in a HMGB1-dependent manner to improve colorectal cancer progression (Yang et al. 2020). In terms of resistance, both the nuclear and cytoplasmic HMGB1 promote autophagy and inhibit tumor cell apoptosis to induce chemotherapy resistance (Yuan et al. 2020). The role of HMGB1 in ferroptosis is also increasingly reported. Neutrophil extracellular traps mediate cardiomyocyte ferroptosis via the Hippo-Yap pathway to exacerbate doxorubicin-induced cardiotoxicity (Zhao et al. 2024), where HMGB1 acts as an iron apoptosis inducer by upregulating ferroptosis in astrocytes, thereby aggravating the acute injury after ischemia in the brain (Davaanyam et al. 2023). In pancreatic ductal adenocarcinoma, Targeting the MCP-GPX4/HMGB1 Axis for Effectively Triggering Immunogenic Ferroptosis (Li et al. 2024a, b); N6F11 treatment caused ferroptotic cancer cell death that initiated HMGB1-dependent antitumor immunity mediated by CD8 + T cells (Li et al. 2023a, b). Previous studies have shown that post-translational modifications (i.e., acetylation, phosphorylation, and methylation) of HMGB1 near or within its nuclear localization sequences (NLS) can induce its translocation to the cytoplasm, leading to the subsequent release of HMGB1 during inflammation (Bonaldi et al. 2003; Ito et al. 2007; Kang et al. 2009). In sepsis, YTHDF2 inhibits the release of HMGB1 and alleviates inflammatory responses (Zeng et al. 2023), but no further exploration has been made on the mechanism of YTHDF2 regulating HMGB1 expression; in primary liver cancer, HMGB1 is demethylated by ALKBH5 and recognized by Reader YTHDF2, promoting its degradation (Chen et al. 2021a, b). Here, we demonstrate for the first time that HMGB1 is a target gene of METTL3 and is degraded by METTL3 in a m6A-YTHDF2-dependent manner. Furthermore, we found that the supplementation of HMGB1 could alleviate the inhibition of ferroptosis by METTL3 in pancreatic cancer. Our study reveals that HMGB1 is a target protein of Writer METTL3 and proves that METTL3 regulates HMGB1 in a m6A-dependent manner, thereby regulating ferroptosis. This provides a certain basis for a new treatment direction in pancreatic cancer.

HMGB1 holds significant potential as both a biomarker and therapeutic target in pancreatic ductal adenocarcinoma (PDAC). Its role in tumor progression is well-documented, but its application faces several challenges. HMGB1 lacks enzymatic activity and has a flexible structure, complicating direct drug targeting. Additionally, it interacts with multiple molecules, further increasing the complexity of using it as a single therapeutic target. For HMGB1 detection, methods such as ELISA, Western blotting, immunohistochemistry (IHC), and mass spectrometry are commonly used. These techniques allow for the quantification of HMGB1 in various samples like serum, plasma, and tissue. The ability to measure HMGB1 in bodily fluids suggests its potential as a non-invasive or minimally invasive biomarker. However, challenges such as variability in release under different conditions and the need for standardized protocols must be addressed. Despite these challenges, emerging strategies aim to modulate HMGB1 function indirectly. Blocking downstream signaling pathways mediated by receptors like RAGE and TLR4 shows promise in preclinical PDAC models. Small molecules such as glycyrrhizin and ethyl pyruvate can inhibit HMGB1 release, limiting its extracellular activity. Another approach involves disrupting HMGB1 complexes with other proteins or DNA, potentially offering new therapeutic avenues. Modulating HMGB1 expression at the transcriptional level is another promising strategy, though further exploration is needed. In summary, while HMGB1’s “undruggable” nature presents challenges, integrating these strategies offers hope for developing effective interventions in PDAC. Future research should validate HMGB1’s utility in larger patient cohorts and diverse clinical settings to facilitate its translation from bench to bedside.

In summary, we have elucidated the molecular mechanism underlying METTL3-mediated regulation of ferroptosis and gemcitabine resistance in pancreatic cancer. Our findings demonstrate that elevated expression of METTL3 in pancreatic cancer is partially dependent on O-GlcNAcylation, which is mediated by OGT. The stabilization of METTL3 protein expression through O-GlcNAcylation at the S118 site enhances its interaction with deubiquitinase EIF3H. Moreover, we have discovered that METTL3 promotes the degradation of ferroptosis driver HMGB1 in an m6A-YTHDF2-dependent manner. Additionally, our study reveals that a combination therapy targeting HMGB1, along with gemcitabine and the ferroptosis inducer RSL3, effectively suppresses pancreatic cancer development. However, our study has certain limitations including insufficient exploration of downstream mechanisms involved in regulating ferroptosis and gemcitabine resistance upon O-GlcNAcylation-induced increase in METTL3 stability. Furthermore, clinical data supporting these findings are lacking in our dataset. In future studies, we aim to investigate whether O-GlcNAcylation of METTL3 can regulate its resistance to ferroptosis and gemcitabine treatment while also exploring its impact on the tumor microenvironment and potential modulation of immune checkpoint blockade efficacy through an m6A-dependent mechanism. These efforts will provide valuable insights into the potential clinical application of METTL3 for treating pancreatic cancer and improving treatment outcomes.

## Supplementary Information

Supplementary Material 1: Fig. S1. METTL3 is upregulated in pancreatic cancer and promotes pancreatic cancer cell proliferation, stemness, and gemcitabine resistance. (A): mRNA levels of METTL3 in seven pairs of pancreatic cancer samples. Total RNA was extracted, and qPCR was performed to quantify METTL3 expression. β-actin was used as an internal reference gene for normalization. Data are presented as mean ± SD (*n* = 16). (B): Western blot detection of METTL3 expression. Protein levels were normalized to β-actin as a loading control. Quantification was performed using ImageJ software. (C, D): Cell viability assays using CCK-8 to detect stable overexpression and stable knockdown of METTL3 in (C) PANC1 and (D) BXPC3 cells. Cells were transfected with METTL3-specific sgRNA or overexpression plasmids using CRISPR/Cas9 or Lipofectamine 3000, respectively. Cell viability was measured at 1, 2, 3, 4 and 5 days post-transfection using the CCK-8 assay. Data are presented as mean ± SD (*n* = 3). (E): Quantification of the diameter of cell spheroids in pancreatic cancer cell lines PANC1 and BXPC3 after METTL3 knockout. Spheroid formation was monitored under serum-free culture conditions for 7 days. Images were captured using a light microscope, and spheroid diameter was quantified using ImageJ software. Data are presented as mean ± SD (*n* = 14). (F): Quantification chart of the percentage of CD133 + pancreatic cancer cells after METTL3 knockout. Flow cytometry was used to quantify the CD133 + cell population. Cells were stained with anti-CD133 antibody and analyzed using a BD FACSCalibur flow cytometer. Data are presented as mean ± SD (*n* = 3). ***p < 0.001, **p < 0.01, *p < 0.05, ns *p* > 0.05.

Supplementary Material 2: Fig. S2. The regulation of ferroptosis is mediated by METTL3. (A): KEGG analysis based on the overlapping genes of METTL3 from the top 2000 differential genes ranked in GSE146806 (siNC vs. siMETTL3) and the genes corresponding to the RNA binding peaks bound by METTL3 in GSE132306. Enrichment analysis was performed using the clusterProfiler package in R, and pathways with adjusted *p*-values < 0.05 were considered significant.

Supplementary Material 3: Fig. S3. The inhibition of ferroptosis in pancreatic cancer cells is mediated by METTL3. (A): Representative images and quantification of mitochondrial superoxide levels in BXPC3 cells with stable knockdown or overexpression of METTL3. Superoxide levels were measured using MitoSOX Red dye. Fluorescence intensity was quantified using ImageJ software. Data are presented as mean ± SD (*n* = 3). (B): Measurement of malondialdehyde (MDA) levels in BXPC3 cells with stable knockdown or overexpression of METTL3. MDA content was determined using a thiobarbituric acid reactive substances (TBARS) assay kit according to the manufacturer’s protocol. Data are presented as mean ± SD (*n* = 9). (C): Measurement of glutathione (GSH) levels in BXPC3 cells with stable knockdown or overexpression of METTL3. GSH content was detected using a GSH/GSSG ratio assay kit following the manufacturer’s instructions. Data are presented as mean ± SD (*n* = 9). (D): Measurement and quantification of reactive oxygen species (ROS) levels in BXPC3 cells with stable knockdown or overexpression of METTL3. ROS levels were assessed using the DCFH-DA fluorescent probe. Fluorescence intensity was measured at excitation/emission wavelengths of 488/525 nm. Data are presented as mean ± SD (*n* = 3). (E): Detection and quantification of ferrous ion levels in BXPC3 cells with stable knockdown or overexpression of METTL3 using FerroOrange dye. Ferrous ion levels were visualized by fluorescence microscopy, and fluorescence intensity was quantified using ImageJ software. Data are presented as mean ± SD (*n* = 3). (F): Measurement of mitochondrial membrane potential in BXPC3 cells with stable knockdown or overexpression of METTL3. Data are presented as mean ± SD (*n* = 3). ***p < 0.001, **p < 0.01, *p < 0.05, ns *p* > 0.05.

Supplementary Material 4: Fig. S4. HMGB1 was identified as a downstream target of METTL3. (A): Quantitative determination of m6A levels in BXPC3 cells with stable knockout and overexpression of METTL3. Total RNA was isolated, and m6A levels were measured using an m6A RNA Methylation Quantification Kit according to the manufacturer’s protocol. Data are presented as mean ± SD (*n* = 9). (B): RIP-qPCR validation of METTL3-regulated HMGB1 m6A modification in BXPC3 cells. METTL3-bound RNA was immunoprecipitated using an anti-METTL3 antibody, and HMGB1 mRNA enrichment was quantified by qPCR. IgG was used as a negative control. Data are presented as mean ± SD (*n* = 3). (C): The m6A level of HMGB1 mRNA when the expression of METTL3 was altered in PANC1 cells, as detected by MeRIP-qPCR. Enrichment of m6A-modified HMGB1 mRNA was quantified by qPCR after immunoprecipitation using an anti-m6A antibody. Data are presented as mean ± SD (*n* = 3). (D): The m6A level of HMGB1 mRNA when the expression of METTL3 was altered in BXPC3 cells, as detected by MeRIP-qPCR. Experimental conditions and analyses were identical to those described in panel C. (E): Protein levels of HMGB1 after knockout or overexpression of METTL3 in PANC1 and BXPC3 cells. Western blot analysis was performed, and protein levels were normalized to β-actin as a loading control. Quantification was performed using ImageJ software. (F): mRNA levels of HMGB1 after knockout or overexpression of METTL3 in PANC1 and BXPC3 cells. Total RNA was extracted, and qPCR was performed to quantify HMGB1 expression. β-actin was used as an internal reference gene for normalization. Data are presented as mean ± SD (*n* = 3). (G): Schematic diagram of luciferase reporter constructs containing wild-type or mutated 3’UTR of HMGB1. The wild-type or mutant versions of HMGB1 3’UTR were cloned into a luciferase reporter plasmid. For mutant versions, two putative N6-methyladenosine-modified adenosines were mutated to cytosines. (H): The m6A level and METTL3 binding level of HMGB1 mRNA when the m6A methylation sites on HMGB1 mRNA were mutated in BXPC3 cells, detected by MeRIP-qPCR. Mutations were introduced into the m6A sites using site-directed mutagenesis. Enrichment of m6A-modified HMGB1 mRNA was quantified by qPCR. Data are presented as mean ± SD (*n* = 3). (I): Mutation of m6A methylation sites on HMGB1 mRNA contributes to the enhancement of HMGB1 mRNA stability in BXPC3 cells. Cells were treated with actinomycin D (5µg/mL) to inhibit transcription, and HMGB1 mRNA levels were measured at different time points (0, 2, 4, 6h) by qPCR. Data are presented as mean ± SD (*n* = 3). ***p < 0.001, **p < 0.01, *p < 0.05, ns *p* > 0.05.

Supplementary Material 5: Fig. S5. Inhibition of ferroptosis by METTL3 and gemcitabine resistance are mitigated by HMGB1. (A): Docking model of HMGB1 and gemcitabine. Molecular docking was performed using AutoDock Vina, and the binding affinity was calculated. Key interacting residues are highlighted. (B): Quantification chart of CD133 + percentage in PANC1 and BXPC3 cells with stable overexpression of METTL3, transfected with or without HMGB1. Flow cytometry was used to quantify the CD133 + cell population. Cells were stained with anti-CD133 antibody and analyzed using a BD FACSCalibur flow cytometer. Data are presented as mean ± SD (*n* = 3). (C): Quantification chart of tumor volume. Tumor volume was measured using calipers every 3 days, and the final volume was recorded at the endpoint of the experiment. (D): Quantification chart of tumor weight. Tumors were excised at the endpoint of the experiment, and their weights were recorded. Data are presented as mean ± SD (*n* = 5). ***p < 0.001, **p < 0.01, *p < 0.05, ns *p* > 0.05.

Supplementary Material 6: Fig. S6. OGT was upregulated in pancreatic cancer. (A): The UALCAN database (https://ualcan.path.uab.edu/index.html) shows that OGT protein is upregulated in pancreatic cancer. The dataset was analyzed using the TCGA Pancreatic Cancer cohort. Statistical significance was determined using a Student’s t-test. Adjusted *p*-values < 0.05 were considered significant.

Supplementary Material 7: Fig. S7. O-GlcNAcylation stabilizes METTL3 protein via enhancing the interaction between METTL3 and EIF3H. (A): Docking model of METTL3 and OGT interaction. Molecular docking was performed using AutoDock Vina, and the binding affinity was calculated. Key interacting residues are highlighted. (B): Interaction area and binding free energy of the relevant complex. Binding free energy was calculated using the MM-PBSA method, and the interaction area was visualized using PyMOL. (C): Truncated variants of OGT. Different domains of OGT were cloned into expression vectors to generate truncated mutants for functional assays. (D): Co-IP assay performed in HEK293T cells transfected with Flag-METTL3 and truncated HA-OGT to detect protein interactions. Cell lysates were immunoprecipitated with anti-Flag antibody, followed by Western blotting with anti-HA and anti-Flag antibodies. (E): Correlation analysis of METTL3 and OGT protein levels in pancreatic cancer. Immunohistochemical analysis was performed on pancreatic cancer tissues, and staining intensity was scored. Pearson correlation analysis was used to determine the relationship. (F): Quantification of METTL3 expression levels under corresponding treatments through qPCR experiments. Total RNA was extracted, and qPCR was performed to quantify METTL3 expression. β-actin was used as an internal reference gene for normalization. Data are presented as mean ± SD (*n* = 3). (G): PANC1 cells transfected with MYC-OGT, HA-UB, HA-UB K48, and HA-UB K63 were subjected to protein A/G agarose immunoprecipitation using anti-METTL3 antibodies, followed by HA Western blotting to detect ubiquitination levels. (H): Venn diagram showing METTL3 mass spectrometry results (PXD036899). METTL3-interacting proteins were obtained from the BioGRID database (https://thebiogrid.org/), and all deubiquitinating enzymes (DUBs) were retrieved from the IUUCD database (http://iuucd.biocuckoo.org/). Overlapping gene EIF3H was identified. (I): Correlation analysis of EIF3H and METTL3 expression obtained from GEPIA2 (http://gepia2.cancer-pku.cn/#index). RNA-seq data from TCGA and GTEx cohorts were used for the analysis. Pearson correlation analysis was performed. (J): METTL3 and EIF3H protein expression in pancreatic cancer obtained from the Proteomic Data Commons database (cancer.gov), with correlation analysis plotted. Protein levels were quantified using mass spectrometry data, and Pearson correlation analysis was performed. (K): METTL3-WT or METTL3-S118A mutants were transfected into PANC1 and BXPC3 cells. Flag immunoprecipitation was performed, followed by Western blotting with the corresponding antibodies to assess protein stability. (L): PANC1 cells were transfected with METTL3-WT or METTL3-S118A plasmids. METTL3 ubiquitination was detected by immunoprecipitation using anti-Flag antibodies, followed by Western blotting with anti-Ubiquitin antibody. ***p < 0.001, **p < 0.01, *p < 0.05, ns *p* > 0.05.

Supplementary Material 8: Fig. S8. The promotion of ferroptosis in pancreatic cancer cells is mediated by the S118A mutant of HMGB1. (A): Representative images and quantification of mitochondrial superoxide levels in PANC1 cells with the S118A mutant of HMGB1. Superoxide levels were measured using MitoSOX Red dye. Fluorescence intensity was quantified using ImageJ software. (B): Measurement of malondialdehyde (MDA) levels in PANC1 cells with the S118A mutant of HMGB1. MDA content was determined using a thiobarbituric acid reactive substances (TBARS) assay kit according to the manufacturer’s protocol. (C): Measurement of glutathione (GSH) levels in PANC1 cells with the S118A mutant of HMGB1. GSH content was detected using a GSH/GSSG ratio assay kit following the manufacturer’s instructions. (D): Measurement and quantification of reactive oxygen species (ROS) levels in PANC1 cells with the S118A mutant of HMGB1. ROS levels were assessed using DCFH-DA fluorescent probe. Fluorescence intensity was measured at excitation/emission wavelengths of 488/525 nm. (E): Detection and quantification of ferrous ion levels in PANC1 cells with the S118A mutant of HMGB1 using FerroOrange dye. Ferrous ion levels were visualized by fluorescence microscopy, and fluorescence intensity was quantified using ImageJ software. (F): Measurement of mitochondrial membrane potential in PANC1 cells with the S118A mutant of HMGB1. ***p < 0.001, **p < 0.01, *p < 0.05, ns *p* > 0.05.

Supplementary Material 9.

Supplementary Material 10.

## Data Availability

No datasets were generated or analysed during the current study.
