# Supplementary figures and images for: O-GlcNAcylation with ubiquitination stabilizes METTL3 to promoting HMGB1 degradation to inhibit ferroptosis and enhance gemcitabine resistance in pancreatic cancer
Source: Mol Med. 2025 Jun 10;31:228. doi: 10.1186/s10020-025-01285-4 (PMC12153122; doi:10.1186/s10020-025-01285-4)

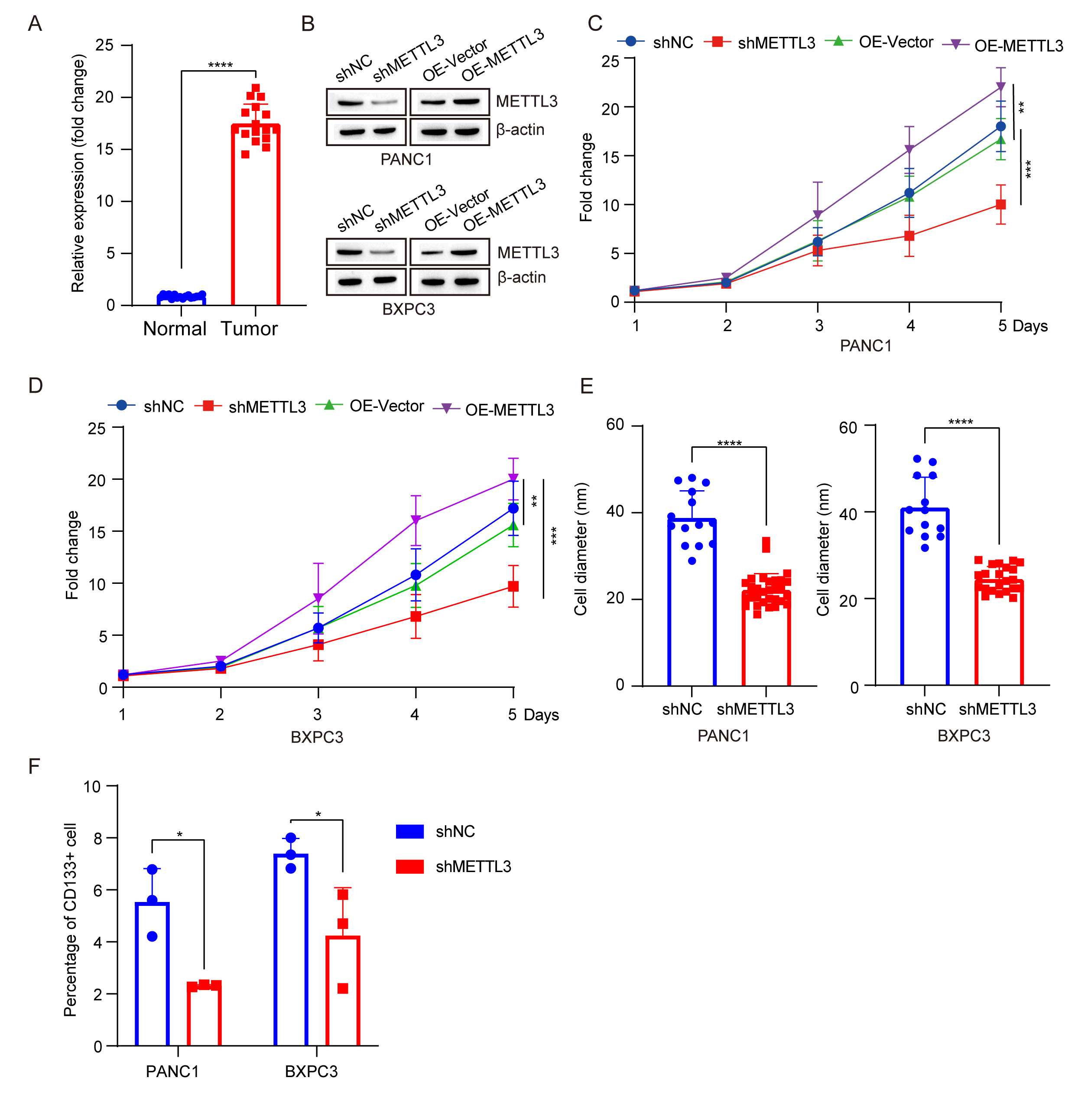

Supplement: Supplementary file 1 — Supplementary Material 1: Fig. S1. METTL3 is upregulated in pancreatic cancer and promotes pancreatic cancer cell proliferation, stemness, and gemcitabine resistance. (A): mRNA levels of METTL3 in seven pairs of pancreatic cancer samples. Total RNA was extracted, and qPCR was performed to quantify METTL3 expression. β-actin was used as an internal reference gene for normalization. Data are presented as mean ± SD (n = 16). (B): Western blot detection of METTL3 expression. Protein levels were normalized to β-actin as a loading control. Quantification was performed using ImageJ software. (C, D): Cell viability assays using CCK-8 to detect stable overexpression and stable knockdown of METTL3 in (C) PANC1 and (D) BXPC3 cells. Cells were transfected with METTL3-specific sgRNA or overexpression plasmids using CRISPR/Cas9 or Lipofectamine 3000, respectively. Cell viability was measured at 1, 2, 3, 4 and 5 days post-transfection using the CCK-8 assay. Data are presented as mean ± SD (n = 3). (E): Quantification of the diameter of cell spheroids in pancreatic cancer cell lines PANC1 and BXPC3 after METTL3 knockout. Spheroid formation was monitored under serum-free culture conditions for 7 days. Images were captured using a light microscope, and spheroid diameter was quantified using ImageJ software. Data are presented as mean ± SD (n = 14). (F): Quantification chart of the percentage of CD133 + pancreatic cancer cells after METTL3 knockout. Flow cytometry was used to quantify the CD133 + cell population. Cells were stained with anti-CD133 antibody and analyzed using a BD FACSCalibur flow cytometer. Data are presented as mean ± SD (n = 3). ***p < 0.001, **p < 0.01, *p < 0.05, ns p > 0.05. [file 10020_2025_1285_MOESM1_ESM.jpg]

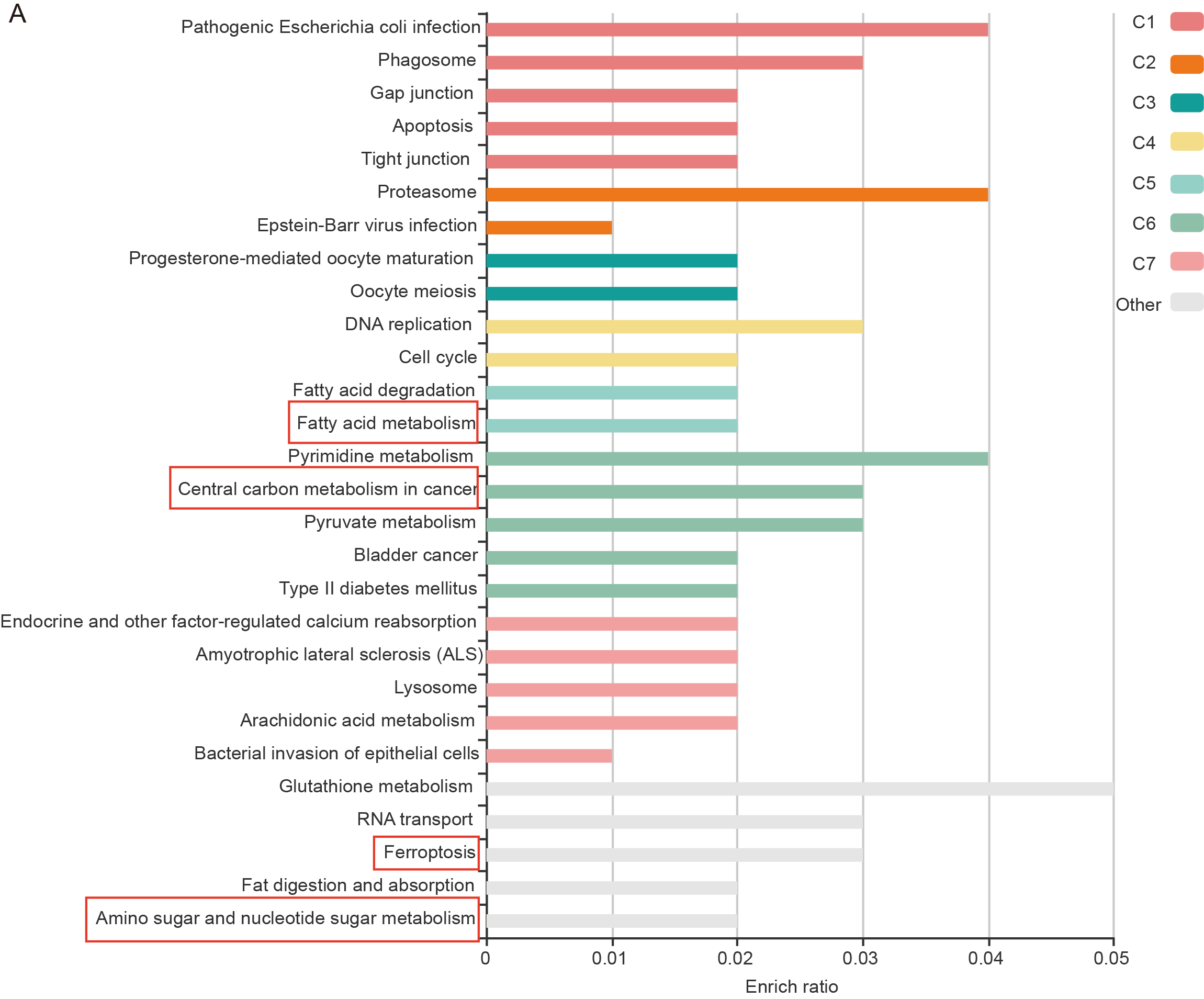

Supplement: Supplementary file 2 — Supplementary Material 2: Fig. S2. The regulation of ferroptosis is mediated by METTL3. (A): KEGG analysis based on the overlapping genes of METTL3 from the top 2000 differential genes ranked in GSE146806 (siNC vs. siMETTL3) and the genes corresponding to the RNA binding peaks bound by METTL3 in GSE132306. Enrichment analysis was performed using the clusterProfiler package in R, and pathways with adjusted p-values < 0.05 were considered significant. [file 10020_2025_1285_MOESM2_ESM.jpg]

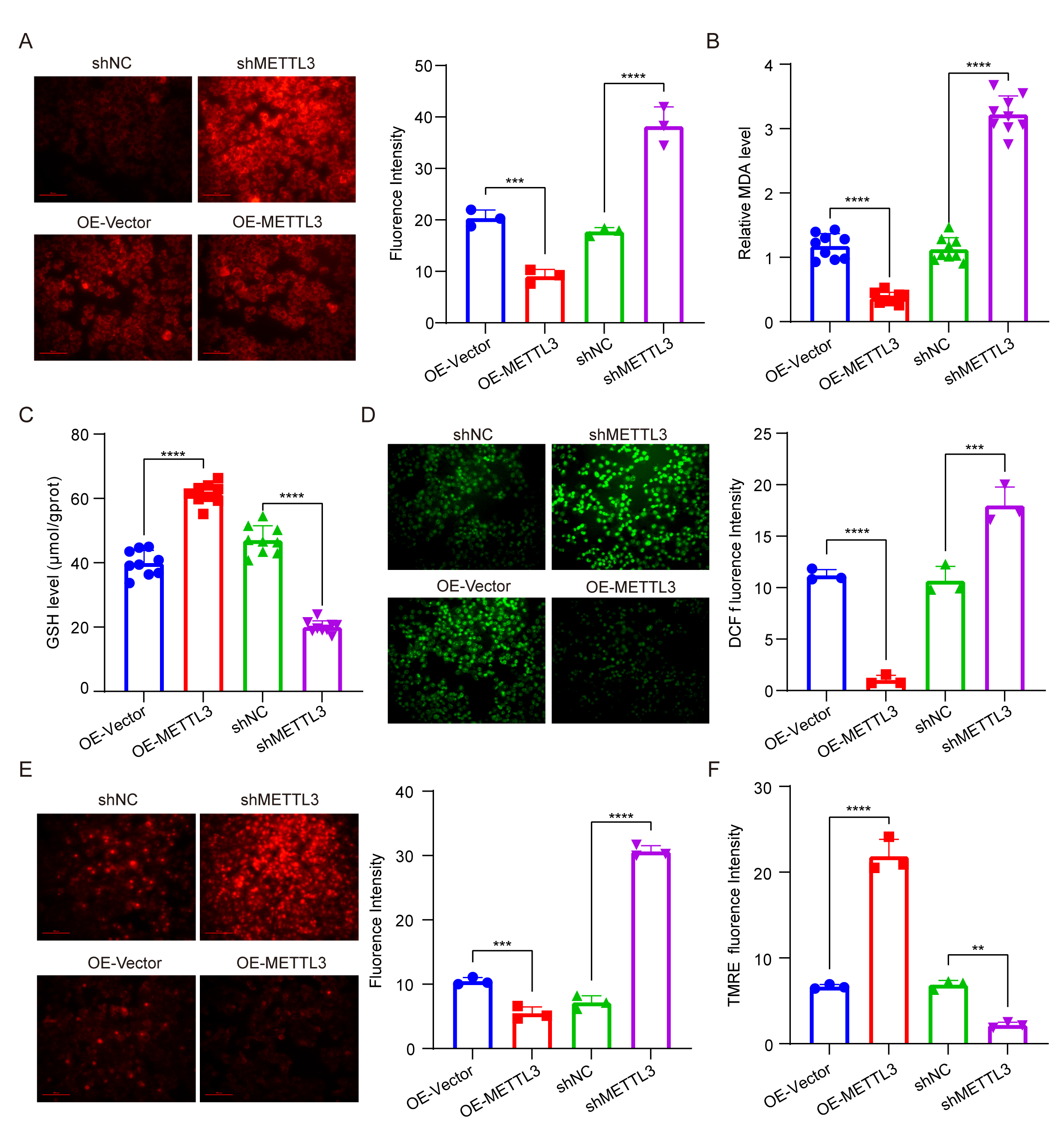

Supplement: Supplementary file 3 — Supplementary Material 3: Fig. S3. The inhibition of ferroptosis in pancreatic cancer cells is mediated by METTL3. (A): Representative images and quantification of mitochondrial superoxide levels in BXPC3 cells with stable knockdown or overexpression of METTL3. Superoxide levels were measured using MitoSOX Red dye. Fluorescence intensity was quantified using ImageJ software. Data are presented as mean ± SD (n = 3). (B): Measurement of malondialdehyde (MDA) levels in BXPC3 cells with stable knockdown or overexpression of METTL3. MDA content was determined using a thiobarbituric acid reactive substances (TBARS) assay kit according to the manufacturer’s protocol. Data are presented as mean ± SD (n = 9). (C): Measurement of glutathione (GSH) levels in BXPC3 cells with stable knockdown or overexpression of METTL3. GSH content was detected using a GSH/GSSG ratio assay kit following the manufacturer’s instructions. Data are presented as mean ± SD (n = 9). (D): Measurement and quantification of reactive oxygen species (ROS) levels in BXPC3 cells with stable knockdown or overexpression of METTL3. ROS levels were assessed using the DCFH-DA fluorescent probe. Fluorescence intensity was measured at excitation/emission wavelengths of 488/525 nm. Data are presented as mean ± SD (n = 3). (E): Detection and quantification of ferrous ion levels in BXPC3 cells with stable knockdown or overexpression of METTL3 using FerroOrange dye. Ferrous ion levels were visualized by fluorescence microscopy, and fluorescence intensity was quantified using ImageJ software. Data are presented as mean ± SD (n = 3). (F): Measurement of mitochondrial membrane potential in BXPC3 cells with stable knockdown or overexpression of METTL3. Data are presented as mean ± SD (n = 3). ***p < 0.001, **p < 0.01, *p < 0.05, ns p > 0.05. [file 10020_2025_1285_MOESM3_ESM.jpg]

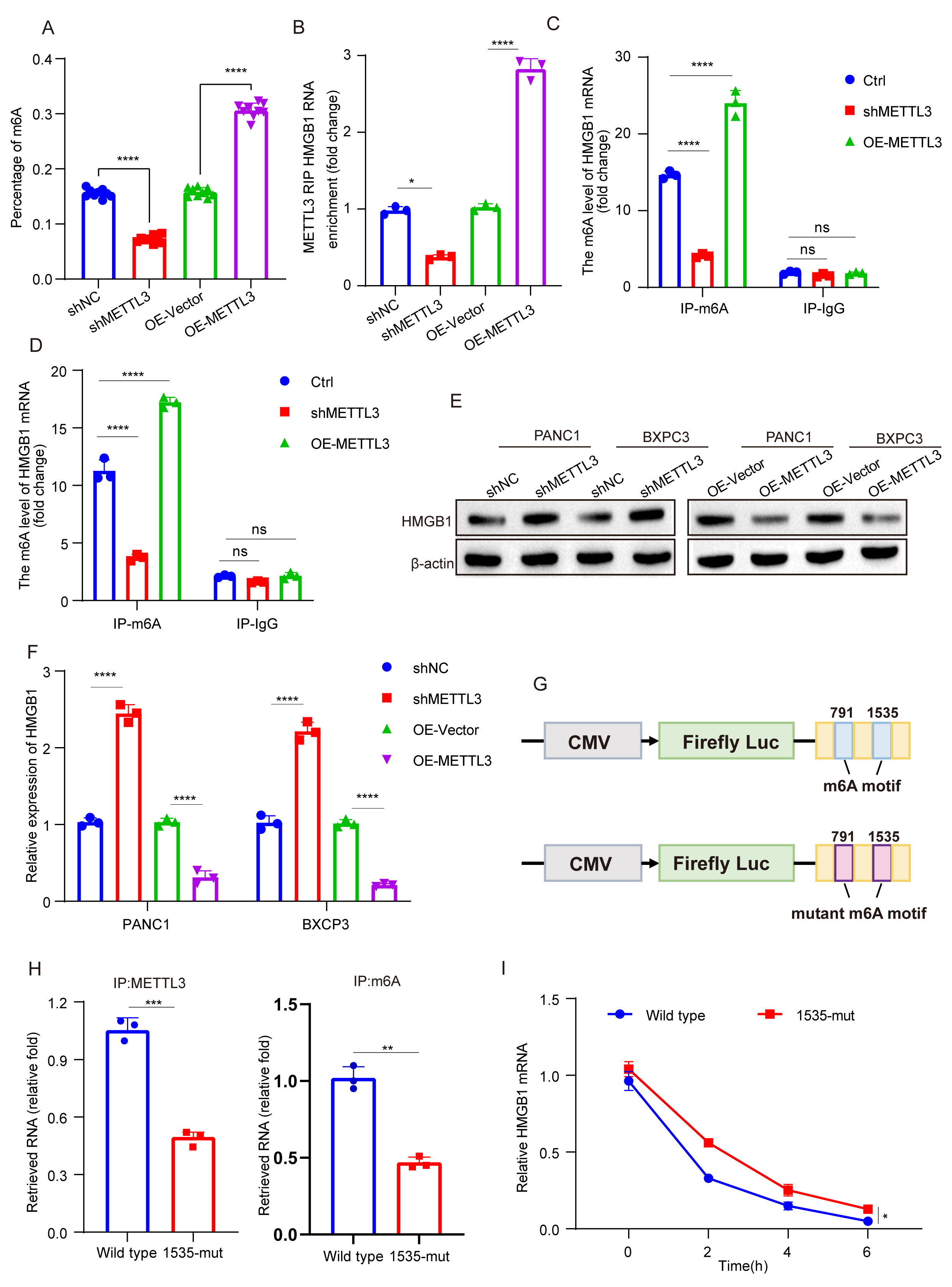

Supplement: Supplementary file 4 — Supplementary Material 4: Fig. S4. HMGB1 was identified as a downstream target of METTL3. (A): Quantitative determination of m6A levels in BXPC3 cells with stable knockout and overexpression of METTL3. Total RNA was isolated, and m6A levels were measured using an m6A RNA Methylation Quantification Kit according to the manufacturer’s protocol. Data are presented as mean ± SD (n = 9). (B): RIP-qPCR validation of METTL3-regulated HMGB1 m6A modification in BXPC3 cells. METTL3-bound RNA was immunoprecipitated using an anti-METTL3 antibody, and HMGB1 mRNA enrichment was quantified by qPCR. IgG was used as a negative control. Data are presented as mean ± SD (n = 3). (C): The m6A level of HMGB1 mRNA when the expression of METTL3 was altered in PANC1 cells, as detected by MeRIP-qPCR. Enrichment of m6A-modified HMGB1 mRNA was quantified by qPCR after immunoprecipitation using an anti-m6A antibody. Data are presented as mean ± SD (n = 3). (D): The m6A level of HMGB1 mRNA when the expression of METTL3 was altered in BXPC3 cells, as detected by MeRIP-qPCR. Experimental conditions and analyses were identical to those described in panel C. (E): Protein levels of HMGB1 after knockout or overexpression of METTL3 in PANC1 and BXPC3 cells. Western blot analysis was performed, and protein levels were normalized to β-actin as a loading control. Quantification was performed using ImageJ software. (F): mRNA levels of HMGB1 after knockout or overexpression of METTL3 in PANC1 and BXPC3 cells. Total RNA was extracted, and qPCR was performed to quantify HMGB1 expression. β-actin was used as an internal reference gene for normalization. Data are presented as mean ± SD (n = 3). (G): Schematic diagram of luciferase reporter constructs containing wild-type or mutated 3’UTR of HMGB1. The wild-type or mutant versions of HMGB1 3’UTR were cloned into a luciferase reporter plasmid. For mutant versions, two putative N6-methyladenosine-modified adenosines were mutated to cytosines. (H): The m6A level an [file 10020_2025_1285_MOESM4_ESM.jpg]

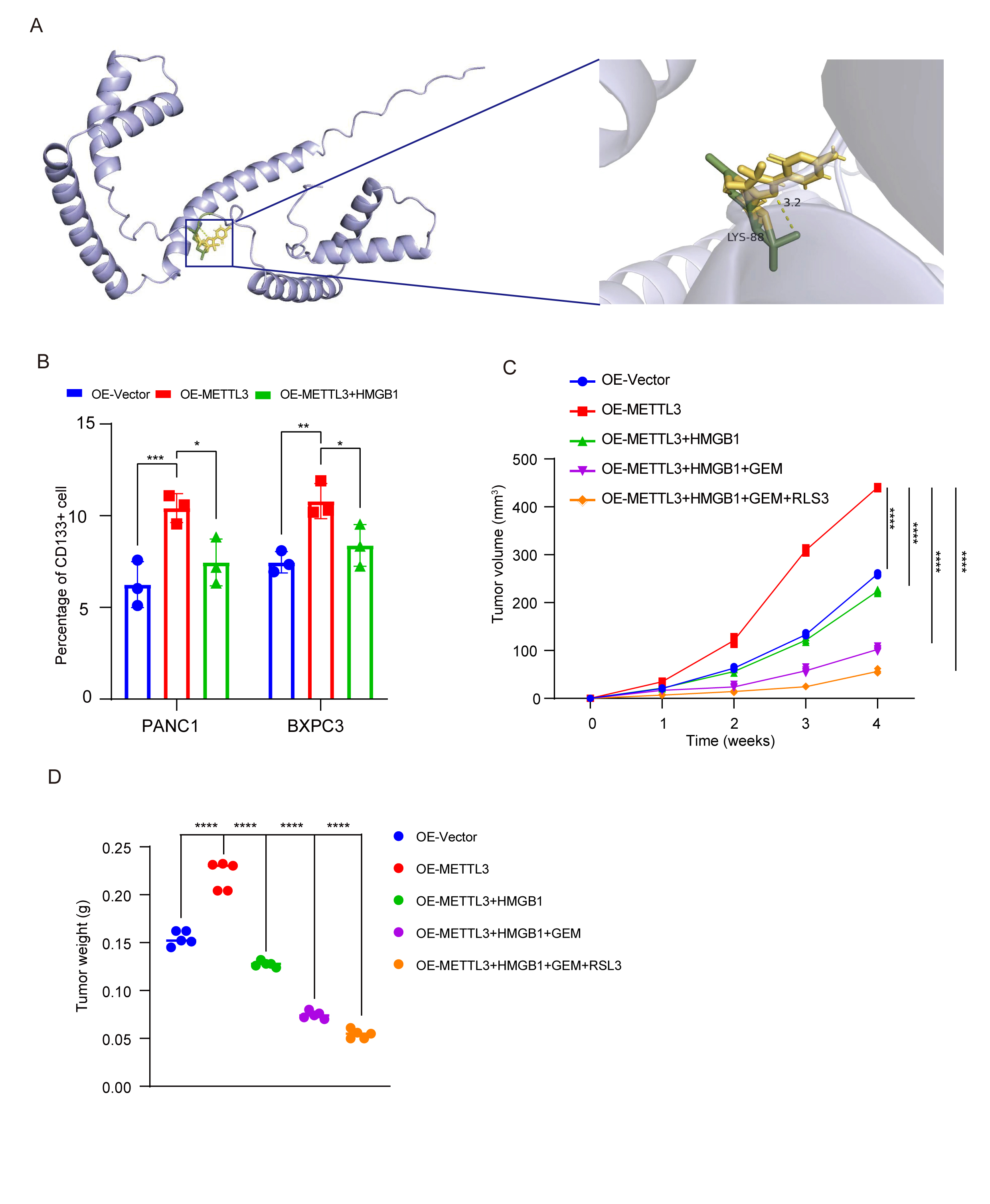

Supplement: Supplementary file 5 — Supplementary Material 5: Fig. S5. Inhibition of ferroptosis by METTL3 and gemcitabine resistance are mitigated by HMGB1. (A): Docking model of HMGB1 and gemcitabine. Molecular docking was performed using AutoDock Vina, and the binding affinity was calculated. Key interacting residues are highlighted. (B): Quantification chart of CD133 + percentage in PANC1 and BXPC3 cells with stable overexpression of METTL3, transfected with or without HMGB1. Flow cytometry was used to quantify the CD133 + cell population. Cells were stained with anti-CD133 antibody and analyzed using a BD FACSCalibur flow cytometer. Data are presented as mean ± SD (n = 3). (C): Quantification chart of tumor volume. Tumor volume was measured using calipers every 3 days, and the final volume was recorded at the endpoint of the experiment. (D): Quantification chart of tumor weight. Tumors were excised at the endpoint of the experiment, and their weights were recorded. Data are presented as mean ± SD (n = 5). ***p < 0.001, **p < 0.01, *p < 0.05, ns p > 0.05. [file 10020_2025_1285_MOESM5_ESM.jpg]

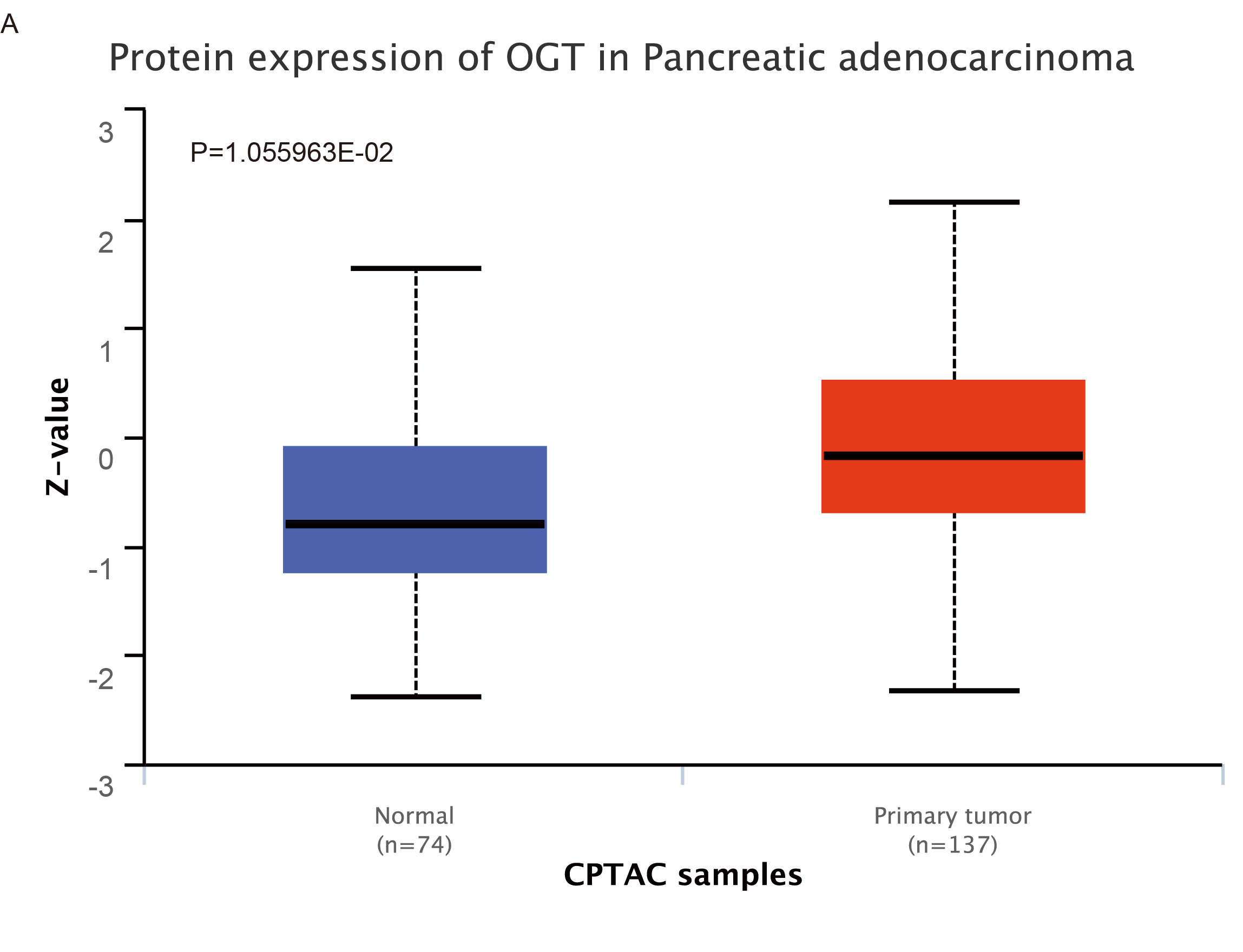

Supplement: Supplementary file 6 — Supplementary Material 6: Fig. S6. OGT was upregulated in pancreatic cancer. (A): The UALCAN database (https://ualcan.path.uab.edu/index.html) shows that OGT protein is upregulated in pancreatic cancer. The dataset was analyzed using the TCGA Pancreatic Cancer cohort. Statistical significance was determined using a Student’s t-test. Adjusted p-values < 0.05 were considered significant. [file 10020_2025_1285_MOESM6_ESM.jpg]

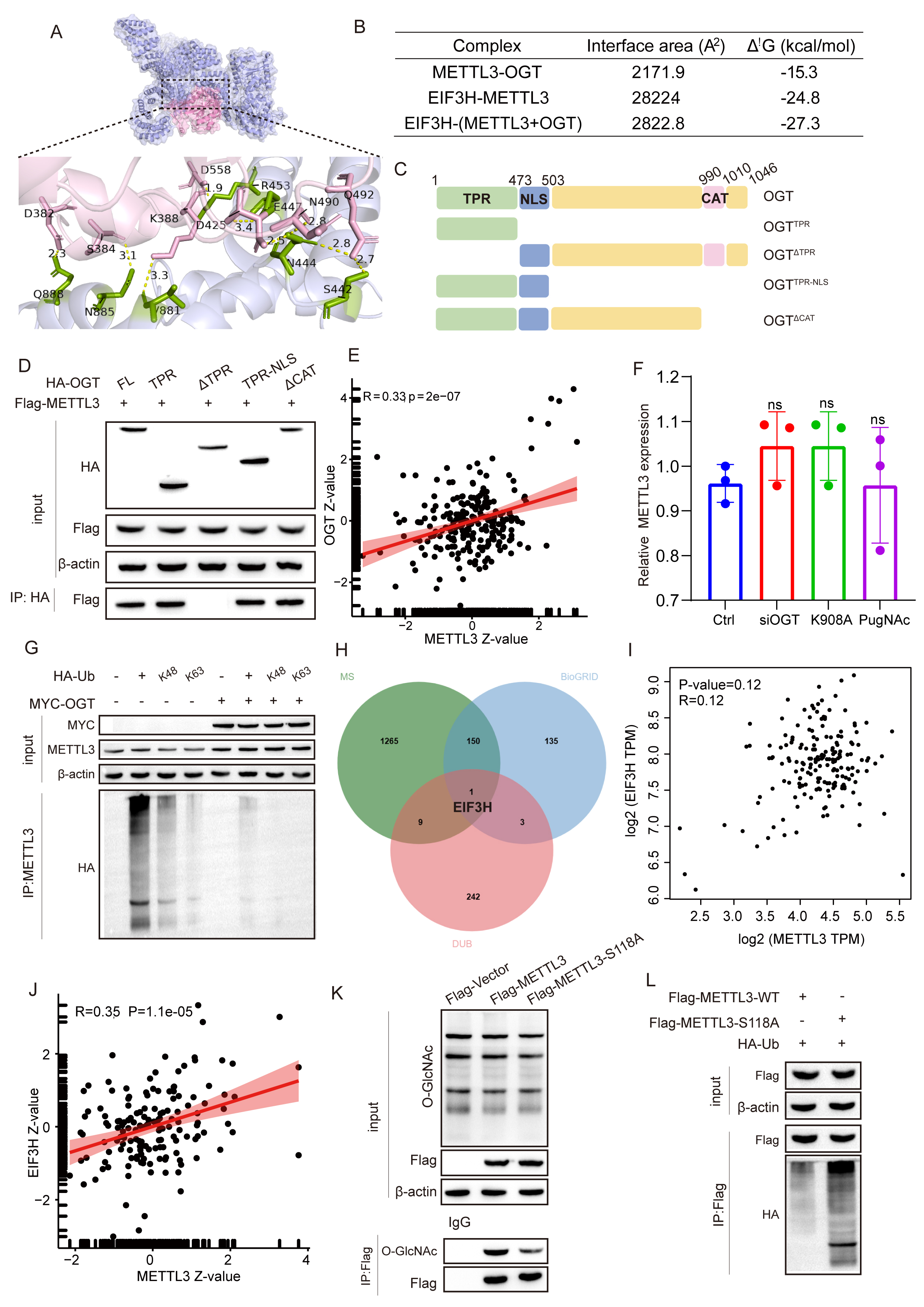

Supplement: Supplementary file 7 — Supplementary Material 7: Fig. S7. O-GlcNAcylation stabilizes METTL3 protein via enhancing the interaction between METTL3 and EIF3H. (A): Docking model of METTL3 and OGT interaction. Molecular docking was performed using AutoDock Vina, and the binding affinity was calculated. Key interacting residues are highlighted. (B): Interaction area and binding free energy of the relevant complex. Binding free energy was calculated using the MM-PBSA method, and the interaction area was visualized using PyMOL. (C): Truncated variants of OGT. Different domains of OGT were cloned into expression vectors to generate truncated mutants for functional assays. (D): Co-IP assay performed in HEK293T cells transfected with Flag-METTL3 and truncated HA-OGT to detect protein interactions. Cell lysates were immunoprecipitated with anti-Flag antibody, followed by Western blotting with anti-HA and anti-Flag antibodies. (E): Correlation analysis of METTL3 and OGT protein levels in pancreatic cancer. Immunohistochemical analysis was performed on pancreatic cancer tissues, and staining intensity was scored. Pearson correlation analysis was used to determine the relationship. (F): Quantification of METTL3 expression levels under corresponding treatments through qPCR experiments. Total RNA was extracted, and qPCR was performed to quantify METTL3 expression. β-actin was used as an internal reference gene for normalization. Data are presented as mean ± SD (n = 3). (G): PANC1 cells transfected with MYC-OGT, HA-UB, HA-UB K48, and HA-UB K63 were subjected to protein A/G agarose immunoprecipitation using anti-METTL3 antibodies, followed by HA Western blotting to detect ubiquitination levels. (H): Venn diagram showing METTL3 mass spectrometry results (PXD036899). METTL3-interacting proteins were obtained from the BioGRID database (https://thebiogrid.org/), and all deubiquitinating enzymes (DUBs) were retrieved from the IUUCD database (http://iuucd.biocuckoo.org/). Overlapping gene EIF3H was identified. ( [file 10020_2025_1285_MOESM7_ESM.jpg]

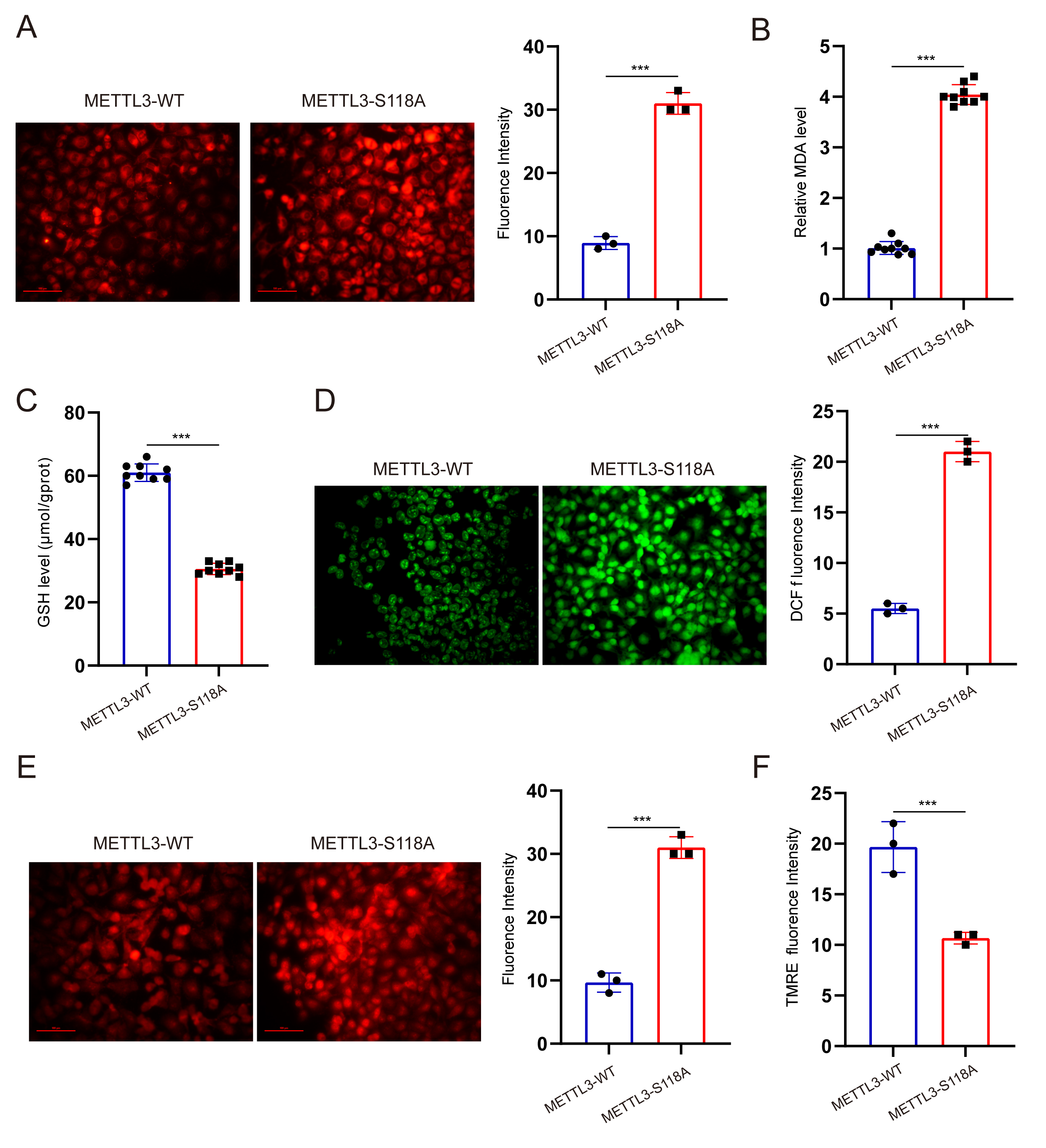

Supplement: Supplementary file 8 — Supplementary Material 8: Fig. S8. The promotion of ferroptosis in pancreatic cancer cells is mediated by the S118A mutant of HMGB1. (A): Representative images and quantification of mitochondrial superoxide levels in PANC1 cells with the S118A mutant of HMGB1. Superoxide levels were measured using MitoSOX Red dye. Fluorescence intensity was quantified using ImageJ software. (B): Measurement of malondialdehyde (MDA) levels in PANC1 cells with the S118A mutant of HMGB1. MDA content was determined using a thiobarbituric acid reactive substances (TBARS) assay kit according to the manufacturer’s protocol. (C): Measurement of glutathione (GSH) levels in PANC1 cells with the S118A mutant of HMGB1. GSH content was detected using a GSH/GSSG ratio assay kit following the manufacturer’s instructions. (D): Measurement and quantification of reactive oxygen species (ROS) levels in PANC1 cells with the S118A mutant of HMGB1. ROS levels were assessed using DCFH-DA fluorescent probe. Fluorescence intensity was measured at excitation/emission wavelengths of 488/525 nm. (E): Detection and quantification of ferrous ion levels in PANC1 cells with the S118A mutant of HMGB1 using FerroOrange dye. Ferrous ion levels were visualized by fluorescence microscopy, and fluorescence intensity was quantified using ImageJ software. (F): Measurement of mitochondrial membrane potential in PANC1 cells with the S118A mutant of HMGB1. ***p < 0.001, **p < 0.01, *p < 0.05, ns p > 0.05. [file 10020_2025_1285_MOESM8_ESM.jpg]
