# Supplementary material for: O-GlcNAcylation with ubiquitination stabilizes METTL3 to promoting HMGB1 degradation to inhibit ferroptosis and enhance gemcitabine resistance in pancreatic cancer
Source: Mol Med. 2025 Jun 10;31:228. doi: 10.1186/s10020-025-01285-4 (PMC12153122; doi:10.1186/s10020-025-01285-4)
Supplement: Supplementary file 9 — Supplementary Material 9. [file 10020_2025_1285_MOESM9_ESM.docx]

Sequence of primer sets

| Primer Name | Primer sequence |
| --- | --- |
| METTL3:forward | 5′-AGCCTTCTGAACCAACAGTCC-3′ |
| METTL3:reverse | 5′-CCGACCTCGAGAGCGAAAT-3′ |
| YTHDF2:forward | 5′-TAGCCAACTGCGACACATTC-3′ |
| YTHDF2:reverse | 5′-CACGACCTTGACGTTCCTTT-3′ |
| HMGB1:forward | 5′ATTCAAGGATCCCAATGCAC-3′ |
| HMGB1:reverse | 5′--GATTTTTGGGCGATACTCAGA-3′ |
| β-actin: forward | 5’-ACCAACTGGGACGACATGGAGAAA-3’ |
| β-actin: reverse | 5’-TAGCACAGCCTGGATAGCAACGTA-3’ |
