# Supplementary material for: O-GlcNAcylation with ubiquitination stabilizes METTL3 to promoting HMGB1 degradation to inhibit ferroptosis and enhance gemcitabine resistance in pancreatic cancer
Source: Mol Med. 2025 Jun 10;31:228. doi: 10.1186/s10020-025-01285-4 (PMC12153122; doi:10.1186/s10020-025-01285-4)
Supplement: Supplementary file 10 — Supplementary Material 10. [file 10020_2025_1285_MOESM10_ESM.docx]

Antibody information

| Antibody | Company | Catalog | dilution rate |
| --- | --- | --- | --- |
| anti-Flag | Sigma | F1804 | 1:1000 |
| anti-O-GlcNAc | Cell Signaling Technology | #9875 | 1:1000 |
| anti-β-actin | Cell Signaling Technology | #4970 | 1:1000 |
| anti-HMGB1 | Cell Signaling Technology | #3935 | 1:1000 |
| anti-eIF3h | Abcam | ab60942 | 1:1000 |
| anti-HA | Santa Cruz biotechnology | sc-57592 | 1:1000 |
| anti-Myc | Proteintech | 60003-2-Ig | 1:1000 |
| anti- METTL3 | Proteintech | 15073-1-AP | 1:1000 |
| anti-YTHDF2 | Proteintech | 24744-1-AP | 1:1000 |
